# Translating Proteomic Into Functional Data: An High Mobility Group A1 (HMGA1) Proteomic Signature Has Prognostic Value in Breast Cancer* [Fn FN2]

**DOI:** 10.1074/mcp.M115.050401

**Published:** 2015-11-02

**Authors:** Elisa Maurizio, Jacek R. Wiśniewski, Yari Ciani, Angela Amato, Laura Arnoldo, Carlotta Penzo, Silvia Pegoraro, Vincenzo Giancotti, Alberto Zambelli, Silvano Piazza, Guidalberto Manfioletti, Riccardo Sgarra

**Affiliations:** From the ‡Department of Life Sciences, University of Trieste, 34127 Trieste, Italy;; §Biochemical Proteomics Group, Department of Proteomics and Signal Transduction, Max-Planck-Institute of Biochemistry, Am Klopferspitz 18, 82152 Martinsried, Germany;; ¶Laboratorio Nazionale CIB, (LNCIB), Area Science Park, 34149 Trieste, Italy;; ¶¶Laboratory of Experimental Oncology and Pharmacogenomics IRCCS - Salvatore Maugeri Foundation, 27100 Pavia, Italy;; ‖Department of Medical Oncology, Hospital Papa Giovanni XXIII, 24127 Bergamo, Italy

## Abstract

Cancer is a very heterogeneous disease, and biological variability adds a further level of complexity, thus limiting the ability to identify new genes involved in cancer development. Oncogenes whose expression levels control cell aggressiveness are very useful for developing cellular models that permit differential expression screenings in isogenic contexts. HMGA1 protein has this unique property because it is a master regulator in breast cancer cells that control the transition from a nontumorigenic epithelial-like phenotype toward a highly aggressive mesenchymal-like one. The proteins extracted from HMGA1-silenced and control MDA-MB-231 cells were analyzed using label-free shotgun mass spectrometry. The differentially expressed proteins were cross-referenced with DNA microarray data obtained using the same cellular model and the overlapping genes were filtered for factors linked to poor prognosis in breast cancer gene expression meta-data sets, resulting in an HMGA1 protein signature composed of 21 members (HRS, HMGA1 reduced signature). This signature had a prognostic value (overall survival, relapse-free survival, and distant metastasis-free survival) in breast cancer. qRT-PCR, Western blot, and immunohistochemistry analyses validated the link of three members of this signature (KIFC1, LRRC59, and TRIP13) with HMGA1 expression levels both *in vitro* and *in vivo* and wound healing assays demonstrated that these three proteins are involved in modulating tumor cell motility. Combining proteomic and genomic data with the aid of bioinformatic tools, our results highlight the potential involvement in neoplastic transformation of a restricted list of factors with an as-yet-unexplored role in cancer. These factors are druggable targets that could be exploited for the development of new, targeted therapeutic approaches in triple-negative breast cancer.

Breast cancer is a striking example of tumor heterogeneity. This feature is one of the main factors impairing the accurate prognosis and prediction of systemic therapies response, a fact that is particularly evident for triple-negative breast cancers (TNBC)^1^ (1). Estrogen and progesterone receptor (ER and PR), HER2/ERBB2, and Ki67 expression levels together with tumor–node-metastasis (TNM) staging are the parameters used to stratify patients and guide therapeutic decisions (2). With the advent of the genomic era, microarray gene expression profile analyses have enabled the categorization of breast cancer into seven main molecular subtypes (luminal A, luminal B, basal-like, Her2-enriched, normal breast-like, claudin-low, and molecular apocrine) (3). In addition to classification, microarray data have been used to search for multigene classifiers constituting molecular signatures with prognostic and predictive value, and these data can also provide a deeper understanding of the multiple interconnected alterations occurring during cell transformation (3). However, the biological heterogeneity of samples often leads to an intrinsic difficulty in identifying those genes that are relevant for cancer biology because the 'real' tumor-driving genes may be hidden in the highly variable individual tumor profiles. Indeed, most of the differentially expressed genes that have been identified using microarray-based gene expression profiling studies of patients' tissues can be classified as “passenger signals,” *i.e.* genes whose expression has been altered as a consequence of the high genomic instability of the cancer cells but that are not directly involved in the development of the disease (4). This finding highlights the need to use biological models when possible to compare the pathological condition *versus* the normal one in the same molecular context to determine genes directly linked to well-defined cancer development stages, which play a role in cell transformation and could constitute more robust and accurate biomarkers (5).

HMGA1 (high mobility group A1) proteins, including HMGA1a and HMGA1b, are architectural transcriptional factors derived from the alternative splicing of the *HMGA1* gene, whose high expression has been demonstrated to be a hallmark of cancer cells (6) and show diagnostic and prognostic value in several cancers (7). Indeed, these proteins have been shown to be relevant “hub proteins” with a well-defined oncogenic role in cells of different origin (7, 8). In regards to breast cancer, several experimental results have assigned a critical role for HMGA1 in driving breast cell transformation. Most human breast cancer cell lines exhibit higher HMGA1 expression levels with respect to nontransformed cell lines (9, 10). A positive correlation between HMGA1 increased expression levels and worse breast cancer clinicopathological features and prognosis has been established (11, 12); however, there are also contrasting data showing that in *BRCA2*-mutated patients, HMGA1 expression is a good prognostic factor for breast cancer outcome (13). Moreover, an alteration of HMGA1 expression levels leads to relevant changes in the tumorigenic properties of breast cancer cell lines. Indeed, HMGA1 over-expression in nonaggressive, nontumorigenic human breast epithelial cells leads to the acquisition of a transformed and aggressive phenotype (10), whereas HMGA1 silencing in highly aggressive, metastatic human breast cancer cell lines leads to reversion of the tumorigenic phenotype, as assessed both by *in vitro* and *in vivo* approaches (9, 12, 14, 15).

HMGA1 proteins exploit pleiotropic mechanisms to drive breast cancer development and progression. Genome-wide approaches performed on breast cancer cell lines clearly demonstrated that HMGA1 proteins influence the expression of migration- and stemness-related genes, as well as genes involved in cell proliferation, epithelial-mesenchymal transition (EMT), and development (10, 12, 15), according to the well-established activities of HMGA1 in neoplastic transformation in general (6). Moreover, several experimental results have highlighted the specific molecular mechanisms influenced by HMGA1 in mammary cell transformation. For instance, HMGA1 proteins inhibit apoptosis by interfering with p53 function (16), downregulate the DNA repair protein BRCA1 (17, 18), interfere with nucleotide excision repair (19), enhance Ras-Raf-MEK-ERK and insulin receptor signaling (20, 21), interfere with the Hippo pathway by promoting YAP nuclear localization (22), and regulate the transcription of miRNAs, such as miRNA-181b, involved in cell cycle control (23). We previously showed that suppression of HMGA1 expression in highly aggressive, MDA-MB-231 TNBC cells led to reversion of the tumoral phenotype. Indeed, HMGA1-silenced cells acquired an epithelial morphology and cell-cell contact inhibition, lost self-renewal capacity, and showed reduced migration, invasion, and metastatic abilities with respect to control cells (12). Therefore, this unique cellular model enables the comparison of gene and protein expression profiles of two well-defined conditions, *i.e.* aggressive *versus* nonaggressive breast cancer cells, in an almost isogenic molecular context, thereby minimizing “passenger signal” changes and underlining those genes that are strictly related to the transformation process itself (4).

In this study, by performing shotgun label-free quantitative proteomics and merging these data with those previously obtained by gene array (hereafter siHMGA1 data set) (12), we determined an HMGA1-linked protein molecular signature composed of 21 factors with prognostic value in breast cancer. Among these 21 factors, we focused on three proteins (KIFC1, LRRC59, and TRIP13) whose involvement in cancer is largely unknown. We demonstrated (1) that their expression is linked to HMGA1, both *in vitro* and *in vivo*; (2) that their gene expression levels have prognostic value in terms of overall, relapse-free, and distant metastasis-free survival (OS, RFS, and DMFS, respectively); and (3) that suppression of their expression in the MDA-MB-231 TNBC cell line significantly impacts cell motility, suggesting an unexplored role for these proteins in cancer invasion and metastasis.

## EXPERIMENTAL PROCEDURES

### 

#### 

##### Cell Culture and Treatments

Silencing experiments in MDA-MB-231 cells were performed as previously described (12). Briefly, MDA-MB-231 cells were grown in Dulbecco's modified Eagle's medium (DMEM) containing 100 U/ml penicillin, 100 μg/ml streptomycin, 2 mm l-glutamine and 10% tetracycline-free FBS (Euroclone S.p.A., Pero (MI), Italy, cat. ECS0182L). The cells were plated at ∼20–30% confluence. After 24 h, silencing was performed upon transfecting the cells with siRNAs (HMGA1 siRNA - siA1_1 and siA1_3 -, KIFC1, LRRC59, TRIP13 siRNA, or control siRNA - siCTRL -, Eurofins MWG Operon, Ebersberg, Germany) using Lipofectamine^TM^ RNAiMAX reagent (Thermo Fisher Scientific/Invitrogen, Waltham MA) for 72 h according to the manufacturer's recommendations. The experiments were performed in biological triplicate. The siRNA sequences are reported in supplemental data. The MDA-MB-231 cell line was kindly provided by Prof. G. Del Sal (Laboratorio Nazionale CIB, (LNCIB), Area Science Park, 34149 Trieste, Italy). Total protein concentrations were quantified after SDS-PAGE analyses followed by Coomassie blue staining and densitometry (Image Scanner, Amersham Biosciences, now GE Healthcare Europe GmbH, Freiburg, Germany Biosciences, Image Master LabScan v.3.00 software). HMGA1 silencing was assessed by quantitative Western blot analyses using an anti-HMGA1 rabbit polyclonal antibody developed in our laboratory. SDS-PAGE and Western blot analyses were performed in accordance with conventional methods.

##### Experimental Design and Statistical Rationale and Proteomic analysis

The cells were washed twice in PBS and lysed in 2% SDS-containing buffer including 100 mm DTT and 100 mm Tris-HCl pH 7.8. Each biological replicate (*n* = 3; HMGA1 silenced cells *versus* control cells, see cell culture and treatments) was analyzed in technical duplicates. The whole cell lysates were processes with MED-FASP using LysC and trypsin (24). Total protein and total peptides were quantified as described previously (25). Liquid chromatographic separation was performed on a C18 reverse phase (12 cm × 75 μm i.d.) column packed with 5 μm resin that was coupled to a LTQ Orbitrap mass spectrometer (Thermo Fisher Scientific, Germany) via a nanoelectrospray source (Proxeon Biosystems, now Thermo Fisher Scientific). The LTQ Orbitrap was operated in data-dependent mode with survey scans acquired at a resolution of 60,000 at m/z 400. For CID fragmentation, as many as 10 of the most abundant precursor ions from the survey scan with a charge ≥ +2 within a 300–1700 *m*/*z* range were selected. The normalized collision energy was 35. The dynamic exclusion parameters were 90 s and 5 ppm. The MS^2^ spectra were acquired in the ion trap. The mass spectrometry (MS) data were analyzed with MaxQuant software (version 1.2.6.20) using the Andromeda search engine (26, 27). The proteins were identified by searching MS and tandem MS (MS/MS) data of peptides against a decoy version of the UniProtKB (May 2013) containing 50,807 sequences. Carbamidomethylation of cysteines was set as a fixed modification. N-terminal acetylation, N-ε-Lysine acetylation, oxidation of methionine, and phosphorylation of serine, threonine, and tyrosine were set as variable modifications. As many as two missed cleavages were allowed. The initial allowed mass deviation of the precursor ion was as high as 6 ppm, and the allowed value for the fragment mass was as high as 0.5 Da. Mass accuracy of the precursor ions was improved by the time-dependent recalibration algorithms of MaxQuant. The “match between runs” option enabled us to match identifications across samples within a time window of 2 min of the aligned retention times. The maximum false peptide discovery rate was specified as 0.01. Label-free quantitation of the data was based on the LFQ intensities (28). Only proteins that were identified in at least four of the samples were subjected to quantitation. Missing values were imputed (width 0.3, downshift 1.8), and the sample data were normalized using the corresponding median values. T-tests were applied for testing differences in protein intensities. Significance of the outliers was calculated by multiple hypothesis testing with a threshold value of 0.05 (29). The mass spectrometry proteomics data have been deposited to the ProteomeXchange Consortium (http://proteomecentral.proteomexchange.org) via the PRIDE partner repository with the data set identifier PXD002032.

##### Lists Overlap

Analysis of the lists of differentially expressed genes and differentially regulated proteins were performed using R. The list of the 21 coregulated proteins is reported in Fig. 3. The list of differentially regulated proteins was filtered considering the *p* value (<0.05). The list of differentially expressed genes (siHMGA1 data set - GSE35525 (12)) was filtered considering the *p* value (<0.05) and the log_2_ fold change (>1.00, <-1.00).

##### Functional Analysis

Functional analysis was performed using the Ingenuity Pathway Analysis tool (Ingenuity® Systems, www.ingenuity.com), DAVID/EASE tool (30), and Oncomine Pro web tool (31, 32). For Oncomine analysis, we create our custom concept composed by the HRS genes. Then our custom concept was analyzed for differential expression in all available “Cancer *versus* Normal” data sets and for differential expression in all available data sets with clinical outcome information. Functional analysis identified the biological functions/transcriptional regulators that were most significant to the data sets. Transcripts were associated with biological functions/transcriptional regulators in the Ingenuity Knowledge Base. A right-tailed Fisher's exact test was used to calculate a *p* value to determine the probability that each biological function/transcriptional regulator assigned to the data set was because of chance alone.

##### Breast Cancer Data sets, Survival Analysis, and GSA

To obtain a survival-related signature, screening for survival-related genes was performed on collection of gene expression data sets using the Kaplan-Meier plotter web tool (33) (updated at version 2014). To verify the correlation of the signature and breast cancer clinical data, KM curves for the OS, DMFS, and RFS of breast cancer patients, classified according to the expression of differentially regulated proteins after HMGA1 silencing (d-A1 and u-A1), the HMGA1 Reduced Signature (HRS), and the KIFC1, LRRC59, and TRIP13 expression levels, were obtained using the Gene Expression-Based Outcome for Breast Cancer Online web tool (GOBO) (33). The samples were split into two groups according to the quantile expressions of the proposed signatures/proteins.

##### qRT-PCR, Wound Healing Assay, and SDS-PAGE and Western Blot Analyses

These analyses were performed as previously described (12). The primer sequences are reported in supplemental data. The antibodies used in the Western blot analyses were the same as those used for the immunohistochemistry analyses. Wound healing assays were performed on 3 ml cell culture plates with cells at about 80% confluence. Measurements were made calculating the area in the middle part of the wounds selecting as much as possible straight and homogeneous zones. Reference points were used to select starting and ending lines for the area measurements.

##### Human Specimens

Breast cancer tissues were selected from the institutional biobank “B. Boerci” at IRCCS Fondazione Salvatore Maugeri (FSM), where remaining tumor tissues intended for research purposes were collected from human donors. The use of human specimens was approved by the FSM Central Ethic Committee, and informed consent was obtained from all patients. The tumor samples (KBr1–15) were selected based on a histopathological analysis performed by the Unit of Pathology; 15 triple-negative basal-like, G3 breast cancers were selected following the immunohistochemistry analyses. Normal epithelial mammary tissues (NBr) were collected at the surgical margins of each breast cancer tissue as a control.

##### Immunohistochemistry

Immunostaining analyses were performed using 5-μm-thick formalin-fixed, paraffin-embedded tissue sections of the breast cancer specimens. Tissues sections of normal breast from each sample were processed as a control. Epitope retrieval was performed in prewarmed pH 6 retrieval buffer in a warm bath before incubation with rabbit anti-HMGA1 (we used two different antibodies developed in our laboratory that we named homemade 1 and homemade 2, 1:1.000), anti-KIFC1 (ab172620, 1/100 - Abcam, Cambridge, UK; ab117535, 1:200 - Abcam), anti-LRRC59 (PA5–32057, 1:500 - ThermoFisher/Pierce; HPA030827, 1/250 - SigmaAldrich), anti-TRIP13 (HPA005727, 1:200 - Sigma-Aldrich; HPA053093, 1/100 - SigmaAldrich) or negative controls. The tumor sections were incubated with the primary antibody solution overnight at 4 °C. HRP-mediated antigen detection was carried out with the LSAB™Plus/HRP kit. The nuclei were counterstained with hematoxylin. The immunostaining results were analyzed using a DM1000 microscope (Leica Microsystems GmbH, Wetzlar, Germany) equipped with LAS (Leica) Software for image capture and analysis. For the scoring of positive cells, at least fifty randomly selected regions for each slide were analyzed. The samples were considered negative when the staining of the breast tissues displayed the same intensity as their normal counterparts.

## RESULTS

### 

#### 

##### HMGA1-regulated Proteins Detected Using a Label-free LC-MS/MS Approach

**Fig. 1.**
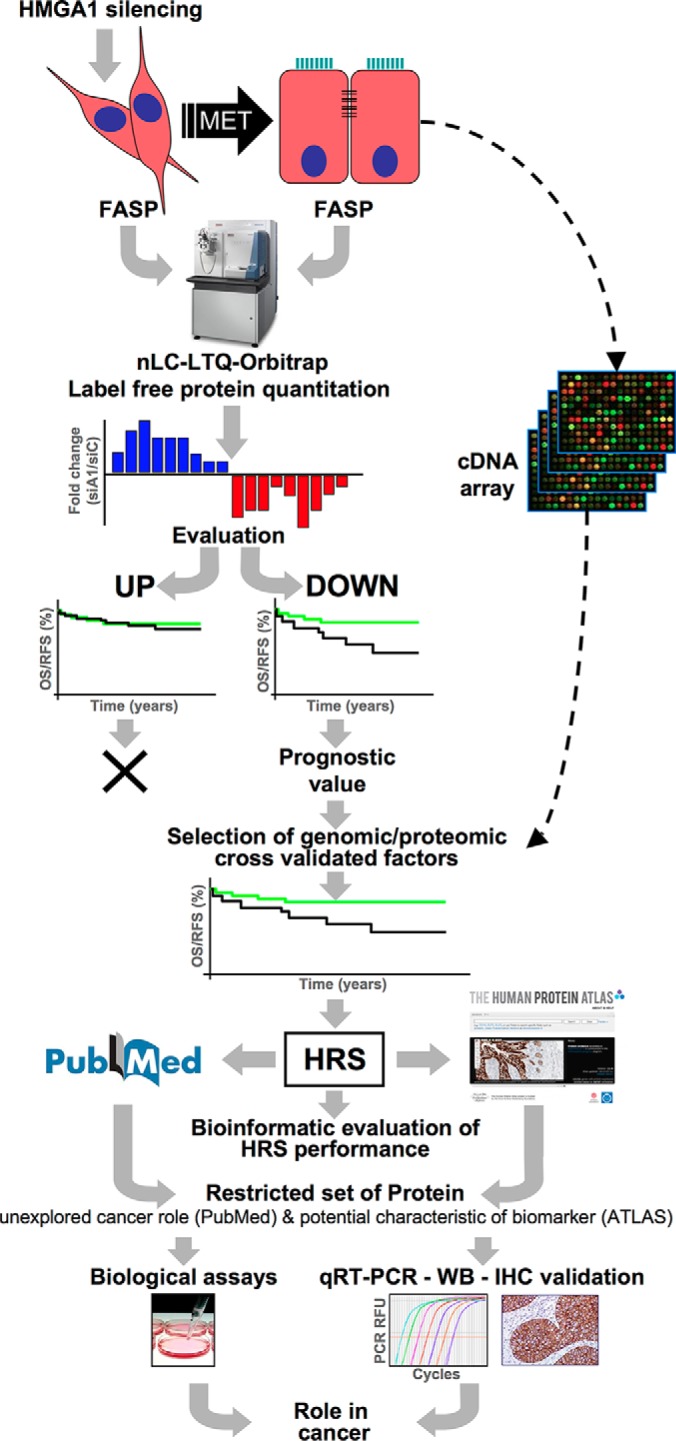
**Schematic representation of the experimental workflow.** Silencing of HMGA1 in MDA-MB-231 breast cancer cells led to a mesenchymal-epithelial transition. The proteins obtained from the control and HMGA1-silenced cells were extracted and treated with the FASP protocol prior to MS analysis and label-free quantitation. The up- and down-regulated sets of proteins were assessed for their prognostic value. Only the downregulated proteins displayed prognostic significance. These proteins were compared with the gene expression analyses performed on the same cellular model and only the common proteins were selected for further analyses. These common proteins were selected on the basis of their prognostic value and resulted in a list of 21 proteins, termed the HMGA1 reduced signature (HRS). Data regarding these 21 proteins were extracted from the PubMed and Human Protein Atlas resources (www.proteinatlas.org), with a focus on those proteins with less cancer-related information and with a differential expression between normal and cancerous tissues.

To gain a deeper view of the proteome-wide changes linked to the loss of aggressiveness caused by HMGA1 silencing (12), we took advantage of high-throughput proteomics based on a label-free shotgun quantitative approach (refer to Fig. 1 for a schematic view of our experimental workflow). HMGA1 proteins were silenced in MDA-MB-231 cells using siRNA. The biological effect of HMGA1 depletion could be observed as the mesenchymal-epithelial morphological transition, which was clearly visible upon comparing HMGA1-silenced (siA1_3) and control (siCTRL) cells (supplemental Fig. S1 and our data reported in (12)). HMGA1 silencing was verified using quantitative Western blot analyses (supplemental Figs. S2 and S3) and turned out to be consistent with our previous reported data (12) The cell lysates (biological triplicate analyses: HMGA1-silenced (siA1_3) *versus* control (siCTRL) cells) were then processed according to the MED-FASP procedure, the peptide mixtures were analyzed using LC-MS/MS on a linear ion trap Orbitrap mass spectrometer (technical duplicate analyses), and the data were processed by MaxQuant. T-tests were applied for testing differences in protein intensities. Significance of the outliers was calculated by multiple hypothesis testing with a threshold value of 0.05 (28). Results of protein identification and quantitation are reported in supplemental Tables S1 and S2.

HMGA1 depletion had a profound impact on the MDA-MB-231 proteome; nearly 17% of the totality of detected proteins (574 out of 3296, among which 292 proteins were down- and 282 were up-regulated, hereafter called d-A1 and u-A1, respectively) displayed significantly altered expression levels. Notably, the expression ratio of HMGA1 in the silenced *versus* control cells, as determined by mass spectrometry, was in accordance with that obtained by quantitative Western blot, thus strengthening the robustness of the MS-based quantitative approaches.

A bird's eye view of the proteomic alterations obtained using bioinformatic pathway analysis tools (IPA and DAVID - Table I and supplemental Tables S3–S5) revealed that the proteomic alterations caused by HMGA1 silencing had a strong effect on cell cycle regulation, chromosome structure, cellular motility mechanism, and protein synthesis. These data are in strong agreement with our preceding data (12).

**Table I:** Biological functions associated with down- and up-regulated genes in HMGA1-silenced MDA-MB-231 cells

Database for Annotation, Visualization and Integrated Discovery (DAVID)		Ingenuity Pathway Analysis (IPA)	
Functional Annotation Clustering		Molecular and Cellular Functions	
DOWN	UP	DOWN	UP
Cell cycle	Protein biosynthesis	Cell cycle	Protein synthesis
Chromosome	Oxidoreductase	Cellular Movement	Gene expression
Nucleotide/ATP binding	Proteasome	Cellular ass. and organiz.	Energy production
Macromolecular complex	Metabolism	DNA repl., recomb. and rep.	Lipid metabolism

##### An HMGA1 Proteomic Signature Has Prognostic Value in Breast Cancer

**Fig. 2.**
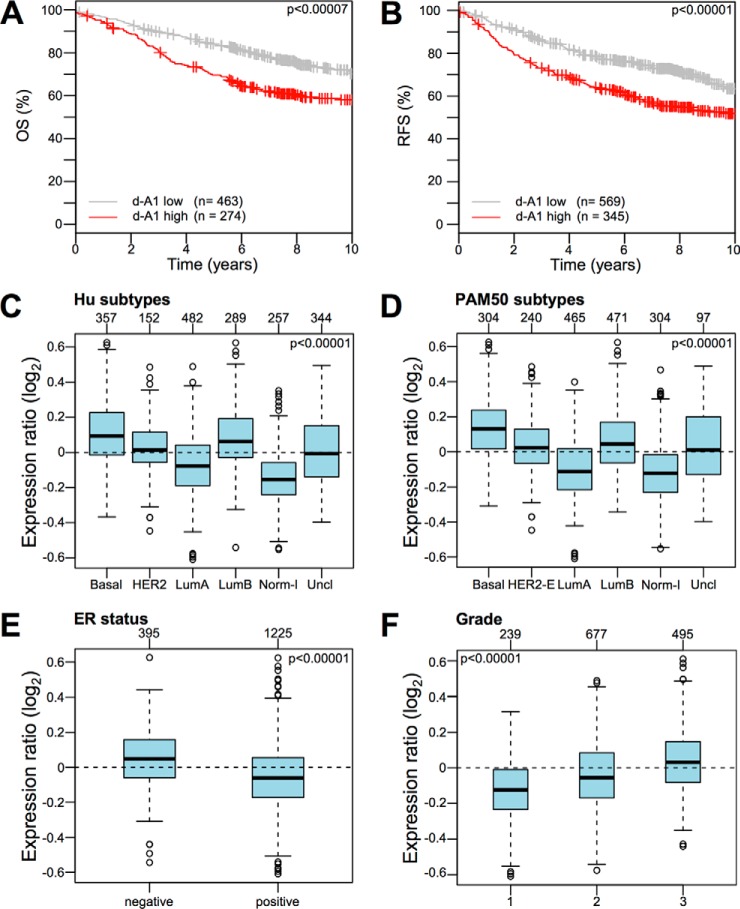
**Proteins whose expression is directly proportional to the HMGA1 expression level (d-A1 protein set) represent a signature associated with the clinical outcome of breast cancer patients and are enriched in specific cancer subtypes.** *A*, *B*, KM plots for OS and RFS with regards to the gene expression level (low or high) in a collection of breast cancer gene expression data sets (Kmplot collection v2014) of the set of proteins that are downregulated following HMGA1 silencing (d-A1 protein set). *C–F*, Gene set analysis (GSA) of the d-A1 protein set expression collection of breast cancer gene expression data sets (GOBO collection v2014). Box plots illustrating the expression distribution of the d-A1 protein set across different cancer subtypes (Hu and PAM50 subtypes), ER-negative and -positive breast cancers, and breast cancers of different histological grade (1, 2, and 3). The numbers above the charts indicate the patients in each subtype group. The boxplots show the mean-centered Log_2_ expression values of the signature across different subtypes/clinical variables.

KM plots obtained upon analyzing a collection of breast cancer gene expression data sets (Kmplot collection v2014) showed that d-A1 proteins represent a molecular signature able to group patients in terms of both OS and RFS (Fig. 2*A* and 2*B*). Moreover, d-A1 proteins were expressed at higher levels in highly aggressive breast cancers, such as basal-like, Her2-enriched, and luminal B subtype (both using HU or PAM50 intrinsic subtypes), as well as ER-negative and grade 3 tumors (Fig. 2*C–2*F). On the contrary, u-A1 proteins did not seem to provide relevant prognostic information and were not enriched in specific breast cancer subtypes (supplemental Fig. S4). Therefore, we focused the bioinformatic and functional analyses on the d-A1 set of proteins (proteins whose expression decreased because of HMGA1 silencing), which are presumed to be positively regulated by HMGA1, either by direct or indirect mechanisms.

The Oncomine web tool, which is a cancer microarray database and web-based data-mining platform aimed at facilitating cancer-related factor discovery from genome-wide expression analyses (32, 33), confirmed that d-A1 proteins were significantly up-regulated in several types of human cancer with respect to normal tissues; moreover, in breast cancer, these proteins were up-regulated in cases with a worse clinical outcome (Table II), further confirming the prognostic value of d-A1. Notably, these bioinformatic data are in concordance both with our previously reported gene array data (12) and with the literature-reported roles for HMGA1 (15, 34). This evidence supports the hypothesis that HMGA1-regulated genes could confer cells an aggressive phenotype, which in turn could be responsible for a worse clinical outcome.

**Table II:** Oncomine analysis of down-regulated proteins (d–A1 protein set)

	Over-/Under-expression in cancer tissues			
	Over	Under	Worst outcome	
			Over	Under
Bladder	5			
Brain and CNS	10	1	5	
Breast	8	1	33	1
Cervical	4			
Colorectal	19		4	1
Oesophageal	2			
Gastric	6			1
Head and Neck	17		1	1
Kidney	3		4	
Leukaemia	1	4	1	1
Liver	4			
Lung	16		9	2
Lymphoma	4		7	8
Melanoma	2		3	
Myeloma	3		5	
Other	14	2	2	
Ovarian	6		5	3
Pancreatic	3		1	
Prostate	2		3	3
Sarcoma	12		1	
Total	140	8	84	21

To obtain a restricted and highly validated list of HMGA1 target proteins, we cross-referenced the proteomic data set with siHMGA1 data set (12). 60 of the 292 (22%) downregulated proteins in our proteomic data set were also downregulated in the siHMGA1 data set. We chose this strategy because protein levels can vary for several reasons, not necessarily linked to mRNA expression levels. Focusing on those genes which show a downregulation both at mRNA and protein levels keeps open the possibility of developing in the future clinical quantitative assays either looking at proteins (*i.e.* IHC) or mRNA (*i.e.* qRT-PCR).

**Fig. 3.**
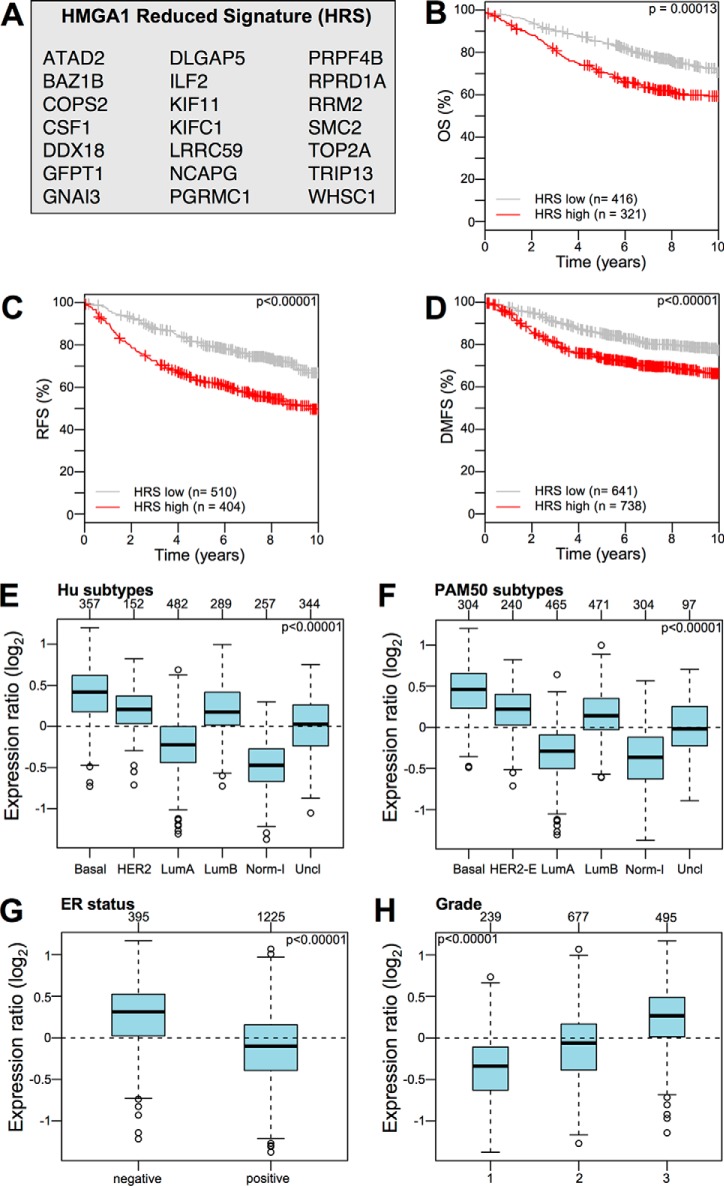
**The HMGA1 reduced signature (HRS) is associated with the clinical outcome of breast cancer patients and is enriched in specific cancer subtypes.** *A*, List of proteins in the HRS. *B–D*, KM plots for OS, RFS, and DMFS with regards to HRS gene expression level (low or high) a collection of breast cancer gene expression data sets (Kmplot collection v2014). *E–H*, GSA of HRS expression collection of breast cancer gene expression data sets (GOBO collection v2014). Box plots illustrating the expression distribution of the HRS across different cancer subtypes (Hu and PAM50 subtypes), ER-negative and -positive breast cancers, and breast cancers of different histological grade (1, 2, and 3). The numbers above the charts indicate the patients in each subtype group. The boxplots show the mean-centered Log_2_ expression values of the signature across different subtypes/clinical variables.

We then performed bioinformatic screening of these 60 proteins for survival-related genes using KM plotter analysis and a manually curated literature inspection, resulting in a list of 21 proteins linked to worse outcomes (RFS), which we referred to as the HMGA1 reduced signature (HRS, Fig. 3*A*). The literature inspection highlighted that for 9 members of the HRS (ATAD2, CSF-1, DLGAP5, KIF11, NCAPG, PGRMC1, RRM2, TOP2A, and WHSC1 - all of the acronyms are explicated in the abbreviation list), there are conspicuous data regarding their role in cancer and their potential use as prognostic markers or cancer therapeutic targets (supplemental Table S6).

Moreover, several HRS members were present in breast cancer-associated gene signatures, as evidenced by the data extracted from GeneSigDB, a curated signature database (supplemental Fig. S5). TOP2A (DNA topoisomerase 2-α) and RRM2 (ribonucleoside-diphosphate reductase subunit M2) are noteworthy examples of proteins with both a well-established role in cancer development and prognostic value (35, 36).

These observations led us to hypothesize that the HRS could be strongly enriched in proteins involved in conferring cells a malignant phenotype. Therefore, we decided to evaluate the HRS in two different ways: (1) as a gene signature with prognostic value and (2) as a source for hypothesis-driven experiments to unravel unexplored HMGA1-dependent molecular mechanisms involved in cancer development.

##### The Clinical Performance of the HMGA1 Reduced Signature (HRS)

High-throughput technologies and genome-wide screenings have led to the development and use of multigene assays (MGAs) as tools to aid oncologists in the difficult decision-making process of treating patients with adjuvant therapy. Some of these MGAs have already been included in the major international guidelines for selecting breast cancer treatments, *i.e.* Oncotype DX and MammaPrint, and the scientific community is now waiting for definitive results from ongoing prospective trials regarding their effective clinical value (37); however, promising evidence suggests that these MGAs bring significant benefits both for patients and healthcare providers (38). To be effective, MGAs must be standardized, adoptable by nonspecialized laboratories, and less time consuming than current approaches. Therefore, the general trend is to start from genome-wide data and end up with a restricted list of proteins that can be evaluated in qRT-PCR- or IHC-based assays. Twenty-one proteins obtained using a cross-validated proteomic-genomic approach comprised our HRS. This signature, as shown in Fig. 3, shares the same breast cancer subtype enrichment profile of the entire set of d-A1 proteins and has almost the same prognostic value as the entire set of d-A1 proteins. Interestingly, the HRS as well as d-A1 proteins were identified as independent prognostic factors for OS, DMSF, and RFS (supplemental Table S7), which indicates that our selection process eliminated “passenger signal proteins,” thereby significantly shortening the original protein list.

Several molecular signatures have been defined in the last decade, and each of them can provide useful prognostic information, despite very low gene/protein overlap. A comparative evaluation of the HRS with respect to other molecular signatures (Table III) shows that the HRS overlaps with at best less than 50% of its composition. In other words, our approach led us to obtain a much smaller but quite unique signature, which is not simply a subset of already known breast cancer signatures.

**Table III:** Overlap of selected breast cancer gene signatures with the HRS

Signature	PubMed ID	N genes	Overlap	*p* values	Topic
ABBA	21082037	111	6	1.22 × 10^−11^	Metasignatures
CRAWFORD	18427120	377	8	5.24 × 10^−11^	BRD4 chromatin modifier
WONG-ESC	18397753	335	7	7.84 × 10^−10^	Cancer stem cells
CARTER	16921376	70	4	7.79 × 10^−09^	Chromosomal instability
MA	12714683	30	3	2.18 × 10^−08^	IDC vs. DCIS
WHITFIELD	12058064	587	7	6.26 × 10^−08^	Cell cycle
RHODES	15184677	67	3	5.95 × 10^−07^	Metasignatures
SOTIRIOU-GGI	16478745	90	3	1.95 × 10^−06^	G2 classification
META-PCNA	22028643	129	3	8.21 × 10^−06^	Cell cycle
PAWITAN	16846532	46	2	1.36 × 10^−05^	Intrinsic molecular signature
SAAL	17452630	162	3	2.02 × 10^−05^	PTEN
CHANG	14737219	355	2	5.42 × 10^−03^	TAF
BEN-PORATH-EXP1	18443585	367	2	5.94 × 10^−03^	Cancer stem cells
HUA	19563758	1345	2	1.55 × 10^−01^	ER1 and RARs

##### Translating Proteomic Into Functional Data: The As-yet-unexplored Role of Selected HRS Members in Cancer and Their Potential Use as Breast Cancer Biomarkers

**Fig. 4.**
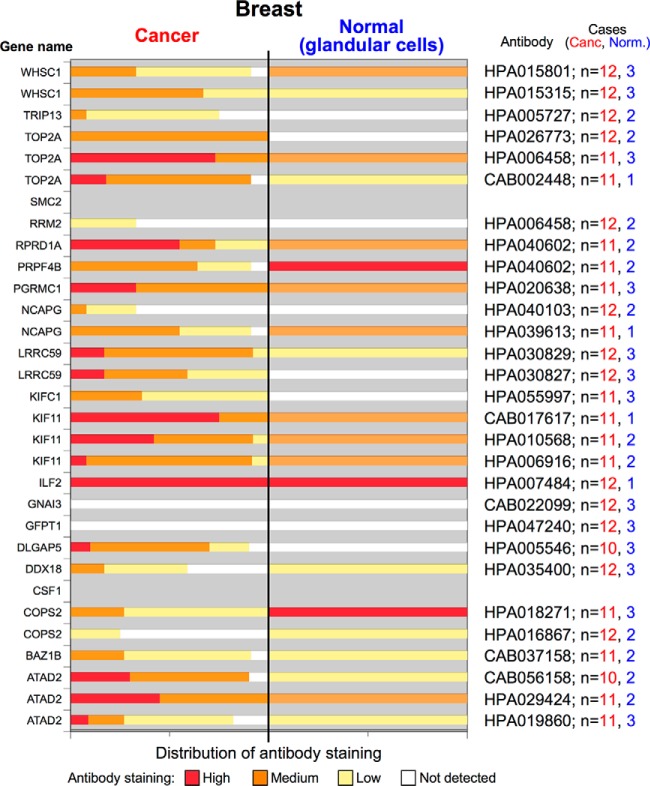
**The expression levels of HRS proteins in breast tissues (cancer *versus* normal) obtained using the publicly available Human Protein Atlas resource.** Each member of the HRS was searched in the Human Protein Atlas resource (cancer tissues - breast cancer; normal tissues - glandular cells), and the percentages of cases showing different levels of antibody staining (high, medium, low, and not detected) are reported as stacked column charts. The antibody identification code together with the number (n) of cancer and normal cases analyzed are reported on the right. Some of the proteins are reported twice or three times according to the number of antibodies used. For CSF1 and SMC2, no data are available.

**Fig. 5.**
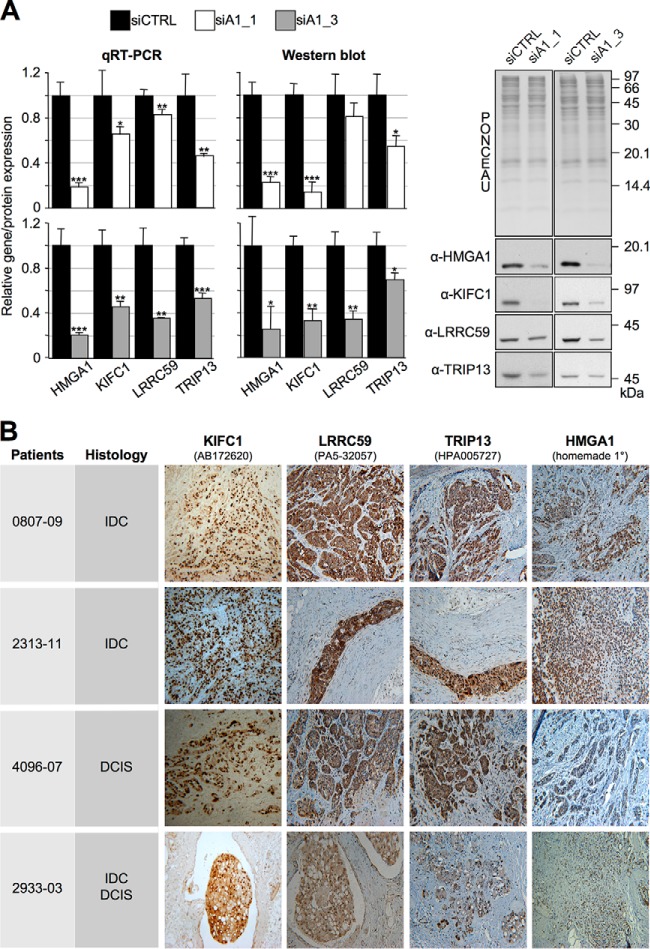
**The expression of KIFC1, LRRC59, and TRIP13 is linked to HMGA1.** MDA-MB-231 cells were treated with control (siCTRL) or HMGA1-targeting siRNAs (siA1_1 and siA1_3) for 72 h. *A*, mRNA and protein expression levels of the indicated genes were analyzed by qRT-PCR and Western blot. Gene expression levels in HMGA1-silenced cells were compared with that of siCTRL cells. GAPDH was used for normalization. Representative WB analyses are shown together with red ponceau stained membranes to verify total protein normalization. The histogram graphs relative to Western blot analyses were obtained using densitometric analyses (siCTRL *versus* siA1_1 and siA1_3). The bars indicate the mean ± S.D. (*n* = 3). Statistical significance was assessed with Student's *t* test (*: *p* < 0.05; **: *p* < 0.01; ***: *p* < 0.001). *B*, Immunohistochemistry analyses performed on breast cancer specimens (IDC, invasive ductal carcinoma; DCIS, ductal carcinoma *in situ*; IDC/DCIS, mixed IDC and DCIS) positive for KIFC1, LRRC59, and TRIP13 and HMGA1.

Genome- and proteome-wide screenings are typically adopted to unravel unexplored functional implications of a specific protein. To exploit our protein list in this direction, we adopted a prioritization criterion based on selecting those components that are less characterized from a cancer-related point of view (see supplemental Table S6) and whose Human Protein Atlas (www.proteinatlas.org) IHC data indicate their use as potential cancer biomarkers because their expression is higher in cancer cells with respect to normal counterparts (Fig. 4). We selected six genes as potential candidates for further exploration, *i.e.* BAZ1B, DDX18, KIFC1, LRRC59, RPRD1A, and TRIP13, and among these, we arbitrarily selected KIFC1, LRRC59, and TRIP13. As a first step, we confirmed that the expression of these three genes was linked to HMGA1 expression levels in MDA-MB-231 cells both by qRT-PCR and western-blot analyses using two different siRNA molecules targeting HMGA1 (Fig. 5*A*). We confirmed these data also in the triple-negative MDA-MB-157 breast cancer cell line (supplemental Fig. S6). Importantly, we confirmed the link between the expression of HMGA1 and KIFC1, LRRC59, and TRIP13 *in vivo* by evaluating their expression in triple-negative G3 breast cancer specimens, either expressing [*n* = 10; 2 invasive lobular carcinoma (ILC), 3 ductal carcinoma *in situ* (DCIS), 4 invasive ductal carcinoma (IDC), and 1 mixed DCIS/IDC] or not expressing HMGA1 proteins [*n* = 5; 5 invasive ductal carcinoma (IDC)]. The results reported in Fig. 5*B* and in supplemental Fig. S7 clearly demonstrate a strong correlation between the expression of HMGA1 and these three proteins. Indeed, in HMGA1-positive specimens, the concordances with HMGA1 expression for the three proteins were as follows: LRRC59, 9/10; KIFC1, 7/7; and TRIP13, 7/10. In HMGA1-negative specimens the concordances were as follows: LRRC59, 4/5; KIFC1, 3/4; and TRIP13, 5/5. Overlapping results were obtained using a different set of antibodies on the same tissue specimens (supplemental Fig. S8 - concordance in HMGA1-positive samples: LRRC59, 7/7; KIFC1, 7/7; TRIP13, 6/7 - concordance in HMGA1-negative samples: LRRC59, 2/4; KIFC1, 3/4, TRIP13, 4/4). Western blot analyses assessing the specificities of the used antibodies are reported in supplemental Fig. S9 and S10.

**Fig. 6.**
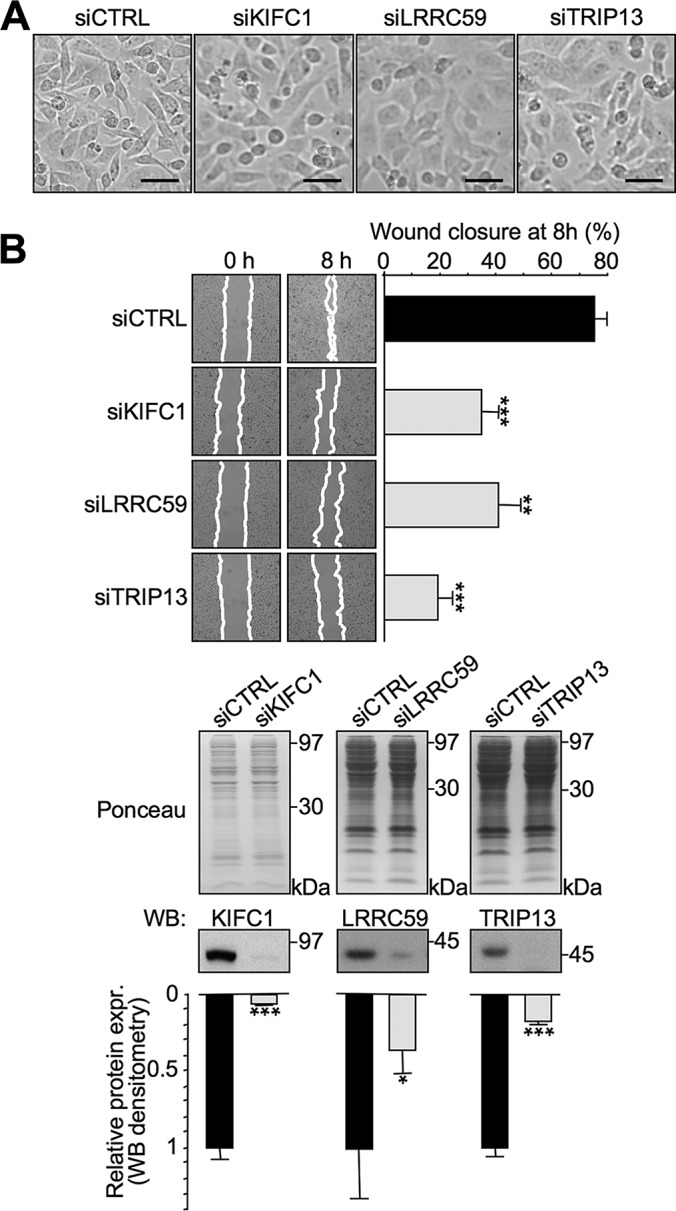
**The silencing of KIFC1, LRRC59, and TRIP13 causes a mesenchymal-epithelial morphological transition in MDA-MB-231 cells that is accompanied by a strong impairment of cell motility.** MDA-MB-231 cells were treated with control siRNA (siCTRL) or siRNA targeting KIFC1, LRRC59, and TRIP13 (siKIFC1, siLRRC59, and siTRIP13). After 72 h, the cells were evaluated for their morphology (optical microscope) (*A*) and motility using wound healing assays (*B*). The scale bar represents 50 μm. The bars indicate the mean ± S.D. (*n* = 3, each point is a technical duplicate). Statistical significance was assessed with Student's *t* test (*: *p* < 0.05; **: *p* < 0.01; ***: *p* < 0.001). The evaluation of silencing efficacy was performed by lysing the cells in SDS-sample buffer after the wound healing assay and analyzing the expression of the three proteins by Western blot. Representative WB analyses are shown together with red ponceau stained membranes to verify total protein normalization. Relative protein expression levels were obtained by densitometric analyses of WB analyses (siCTRL *versus* siKIFC1, siLRRC59, and siTRIP13). The bars indicate the mean ± S.D. (*n* = 3). Statistical significance was assessed with Student's *t* test (*: *p* < 0.05; **: *p* < 0.01; ***: *p* < 0.001).

**Fig. 7.**
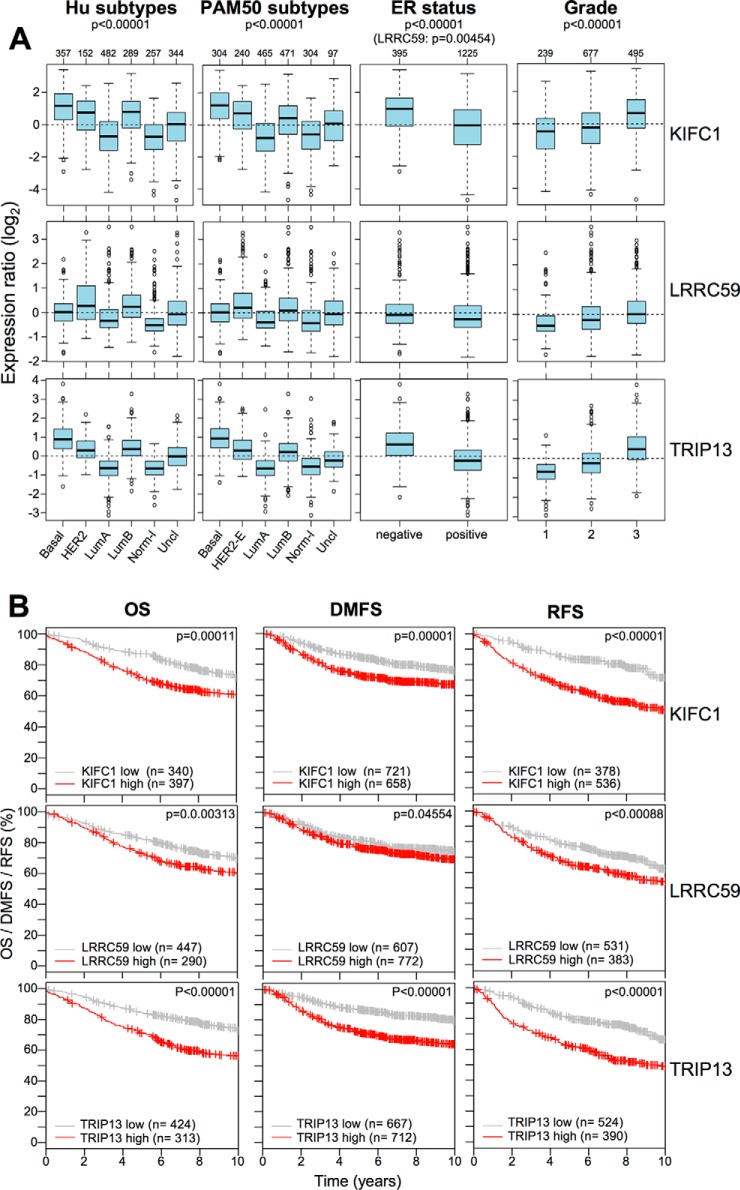
**KIFC1 and TRIP13 are associated with the clinical outcome of breast cancer patients and are enriched in specific cancer subtypes.** *A*, GSA of KIFC1, LRRC59, and TRIP13 expression in collection of breast cancer gene expression data sets (GOBO collection v2014). Box plots illustrate the expression distribution of KIFC1, LRRC59, and TRIP13 across different cancer subtypes (Hu and PAM50 subtypes), ER-negative and -positive breast cancers, and breast cancers of different histological grade (1, 2, and 3). The numbers above the charts indicate the patients in each subtype group. The boxplots show the mean-centered Log_2_ expression values of the signature across different subtypes/clinical variables. *B*, KM plots for OS, RFS, and DMFS with regards to the KIFC1, LRRC59, and TRIP13 gene expression level (low or high) in a collection of breast cancer gene expression data sets (Kmplot collection v2014).

Because one of the main effects attainable by HMGA1 silencing in basal-like TNBC cells is an evident morphological transition from a mesenchymal phenotype toward an epithelial one, typically accompanied by a strong impairment of cell motility (12), we assessed whether KIFC1, LRRC59, and TRIP13 partially contribute to these HMGA1-linked effects in MDA-MB-231 cells. Using siRNA, we silenced the expression of these three genes and performed wound healing assays and evaluated cell morphology in parallel (Fig. 6). As observed in Fig. 6*A*, there was an evident transition from a mesenchymal phenotype toward an epithelial one accompanying each of the three gene silencing experiments. Moreover, the wound healing experiments (Fig. 6*B*) clearly demonstrated that the silencing of KIFC1, LRRC59, and TRIP13 strongly impaired wound closure, thus indicating that these three genes have an impact on cell motility pathways. Noteworthy, the silencing of these three proteins has the same effects in the in the triple-negative MDA-MB-157 breast cancer cell line (supplemental Fig. S11). The acquisition of mesenchymal features and increased cell motility are essential steps during the process of tumor metastasis. Therefore, the experimental results obtained by silencing the expression of these three genes prompted us to evaluate their potential clinical value. By interrogating a breast cancer gene expression data set (GOBO data set), we obtained the expression of KIFC1, LRRC59, and TRIP13 genes for tumor samples stratified according to HU and PAM50 subtypes, ER status, and histological grade. We also evaluated the OS, DMFS, and RFS KM curves (Kmplot data set, v2014). As shown in Fig. 7*A*, KIFC1 and TRIP13 demonstrated more interesting results from a clinical point of view with respect to LRRC59. Indeed, these two genes were expressed at higher levels in the more aggressive subtypes (*i.e.* basal-like, Her2-overexpressing, and luminal B), in ER-negative tumors, and in grade 3 classified tumors. Moreover, KM curves (panel B) clearly indicated that both KIFC1 and TRIP13 outperformed LRRC59 in terms of clinical value with regard to OS, DMFS, and RFS. Furthermore, multivariate analyses considering their expression levels together with size (>20 mm), age (>50 years), grade (G3), node status (negative), and ER status (positive) indicated that they represent independent prognostic factors for OS, DMSF, and RFS and that KIFC1 and TRIP13 outperformed LRRC59 (supplemental Table S8). These findings were further supported by interrogating the Oncomine cancer microarray database for the expression of these three genes in twenty different cancer types (supplemental Table S9). KIFC1 was overexpressed in 42 out of 459 analyses (cancer tissue *versus* normal tissue) and TRIP13 in 69 out of 467 analyses (fold change threshold: 2; *p* value threshold: 1 × 10^−4^). Moreover, the cancer type more enriched for KIFC1 overexpression was found to be breast cancer (11), followed by lung (7) and prostate cancer (4). On the other hand, the cancer type showing more results for TRIP13 overexpression was colorectal cancer (15), followed by lung (9) and sarcoma (7).

## DISCUSSION

Exploiting a label-free shotgun quantitative proteomic approach in which the results were cross-referenced with gene array data, we obtained a list of 60 HMGA1-linked genes downregulated at both the mRNA and protein level in a basal-like TNBC cell line. Following bioinformatic filtering for clinically relevant genes obtained using breast cancer gene expression data sets, we obtained a panel of 21 factors that we referred to as the HMGA1 reduced signature (HRS), whose expression was linked to poor prognosis in cancer. This signature showed prognostic value, demonstrated the validity of our approach in shortening the original molecular signature, and highlighted a multifaceted role of HMGA1 proteins in regulating tumor aggressiveness.

Molecular signatures not only represent a clinical tool to aid clinicians in decision-making processes but can also provide clues that highlight the involvement of new genes in specific pathologies. As often occurs in large-scale screenings to identify molecular signatures with clinically relevant features, many of the genes composing these signatures already have a clear connection with the specific pathology taken into consideration. On the contrary, those genes not yet characterized represent potential novel molecules that could be specifically targeted or that could provide novel insight into the molecular mechanisms of the pathology onset. In this work, we focused on three underexplored proteins (KIFC1, TRIP13, and LRRC59). We demonstrated that these proteins lie downstream of HMGA1 (both in a cellular model and in clinical specimens) and are involved in the modulation of cell motility, which is one of the first characteristics a cancer cell must acquire to disseminate.

• KIFC1 (kinesin family member C1) is a minus end-directed motor protein of the kinesin-14 family, which is involved in the process of centrosome clustering in cancer cells that display amplified centrosomes (39). Very little is known regarding the involvement of this protein with other aspects of cancer progression, albeit that it constitutes a potential prognostic marker for ovarian adenocarcinoma (40) and was found to be associated with metastatic spread to the brain of nonsmall cell lung cancer (41). Notably, a drug targeting KIFC1 has been already developed (AZ82) and tested in cancer cells, and this drug was demonstrated to specifically affect the survival of cancer cells with amplified centrosomes (42). Centrosomes are frequently amplified in cancer cells, leading to mitotic defects that cause karyotypic changes arising from chromosome mis-segregation (43), and centrosome clustering is a stratagem used by cancer cells to limit catastrophic effects of multipolar mitosis that is usually linked to unsustainable levels of aneuploidy; however, centrosome amplification and clustering also fuel cell motility. Indeed, centrosomes are “microtubule organizing centers” that are responsible for the proper organization of the Golgi apparatus during interphase, which in turn provides cells with a directional flux of vesicles involved in the transport of migration promoting factors to the leading edge (44).

• TRIP13 (thyroid hormone receptor interacting protein 13) is an AAA-ATPase involved both in mitotic checkpoint regulation (45) and the regulation of meiotic recombination and chromosome structure development (46). Very recently, it has been demonstrated that TRIP13 overexpression is associated with enhanced nonhomologous end joining (NHEJ) activity, leading to error accumulation and development of chemoresistance (47). TRIP13 also appears in cancer-associated molecular signatures (48, 49), therefore underlining its potential role in cancer.

• LRRC59 (leucine-rich repeat containing 59) is an ER-anchored protein and an intracellular binding partner for fibroblast growth factor 1 (FGF1), and this protein is essential for the noncanonical signaling pathway of FGF1 (50). Indeed, FGF1, in addition to the signaling cascade activated by binding to its high-affinity cell-surface receptors (FGFR1–4), can be translocated via an LRRC59-dependent mechanism directly into the nucleus where it has direct regulatory functions (51).

Consistent with this information and with our experimental evidence, bioinformatic evaluation of the clinical predictive performance of these three genes clearly indicates their connection with a worse outcome of the cancer pathology, especially in regards to KIFC1 and TRIP13.

HMGA1 is a multifunctional protein whose overexpression perturbs different processes leading to neoplastic transformation. This feature, together with the fact that it is highly expressed in cancer cells, but nearly undetectable in normal tissues, suggests that it could be an ideal chemotherapeutic target (52–55); however, the identification of its downstream functional effectors could also provide unexplored opportunities to target cancer cells.

In conclusion, despite intrinsic limitations of this study (*i.e.* data obtained by analyzing one single human triple-negative breast cancer cell line and the lack of true normal breast tissue specimens for the evaluation of protein expression in IHC analyses), our study allowed us to define a short molecular signature linked to HMGA1 expression that has prognostic value in breast cancer and that could be eventually used for developing an RT-PCR based tests. More interestingly, our data allowed to determine a previously unknown role for three proteins in regulating cell motility and possibly the entire process leading to the acquisition of metastatic features. Two of these proteins (KIFC1 and TRIP13) display enzymatic activity, thus opening the possibility to develop specific drugs for targeted therapies. These findings assume relevance particularly in regards to TNBC, a unique and extremely heterogeneous breast cancer form for which cytotoxic chemotherapy still remains a less than ideal option because no targeted therapies are available (1). Moreover, gene expression data sets highlight that the overexpression of these two factors is not limited to breast cancer; therefore, these factors should be further investigated as they may be valuable new potential broad-spectrum biomarkers to be exploited in IHC prognostic evaluations and also as cancer specific targets.

## Supplementary Material

Supplemental Data

## Footnotes

^1^ The abbreviations used are:

TNBC: Triple-negative breast cancer
ATAD2: ATPase family AAA domain-containing protein 2
BAZ1B: Tyrosine-protein kinase BAZ1B
BRCA1: Breast cancer type 1 susceptibility protein
BRCA2: Breast cancer type 2 susceptibility protein
COPS2: COP9 signalosome complex subunit 2
CSF1: Macrophage colony-stimulating factor 1
d-A1: Downregulated proteins in HMGA1 silenced MDA-MB-231 cells
DCIS: Ductal carcinoma in situ
DDX18: ATP-dependent RNA helicase DDX18
DLGAP5: Disk large-associated protein 5
DMFS: Distant metastasis-free survival
EMT: Epithelial-mesenchymal transition
ER: Oestrogen receptor
GFPT1: Glutamine-fructose-6-phosphate aminotransferase [isomerizing] 1
GNAI3: Guanine nucleotide-binding protein G(k) subunit alpha
HER2/ERBB2: Human epidermal growth factor receptor 2
HMGA: High mobility group A
HRS: HMGA1 reduced signature
IDC: Invasive ductal carcinoma
ILC: Invasive lobular carcinoma
ILF2: Interleukin enhancer-binding factor 2
Ki67: Antigen identified by monoclonal antibody Ki-67
KIF11: Kinesin-like protein KIF11
KIFC1: Kinesin family member C1
KM: Kaplan-Meier
LRRC59: Leucine rich repeat containing 59
MED-FASP: Multiple enzymes for sample digestion – Filter-aided sample preparation
MGAs: Multigene assays
NCAPG: Condensin complex subunit 3
OS: Overall survival
PGRMC1: Membrane-associated progesterone receptor component 1
PR: Progesterone receptor
PRPF4B: Serine/threonine-protein kinase PRP4 homolog
RFS: Relapse-free survival
RPRD1A: Regulation of nuclear pre-mRNA domain-containing protein 1A
RRM2: Ribonucleoside-diphosphate reductase subunit M2
SMC2: Structural maintenance of chromosomes protein 2
TNM: Tumour-node-metastasis
TOP2A: DNA topoisomerase 2-alpha
TRIP13: Thyroid hormone receptor interactor 13
u-A1: Upregulated proteins in HMGA1 silenced MDA-MB-231 cells
WHSC1: Histone-lysine N-methyltransferase NSD2.
