# Supplementary material for: Translating Proteomic Into Functional Data: An High Mobility Group A1 (HMGA1) Proteomic Signature Has Prognostic Value in Breast Cancer
Source: Mol Cell Proteomics. 2015 Nov 2;15(1):109–23. doi: 10.1074/mcp.M115.050401 (PMC4762532; doi:10.1074/mcp.M115.050401)
Supplement: Supplemental Data [file supp_15_1_109__index.html]

Translating proteomic into functional data: an HMGA1 proteomic signature has prognostic value in breast cancer — Translating Proteomic Into Functional Data: An High Mobility Group A1 (HMGA1) Proteomic Signature Has Prognostic Value in Breast Cancer — A Proteomic HMGA1-linked Signature in Breast Cancer — Supplemental Data 

# Translating Proteomic Into Functional Data: An High Mobility Group A1 (HMGA1) Proteomic Signature Has Prognostic Value in Breast Cancer

## Supplemental Data

- Supplemental data (M&M + Figure legends) (.pdf, 149 KB) - Supplemental Materials and Methods and Figure Legends
- Supplemental Table S1 (.xlsx, 2.6 MB) - Proteomic data I
- Supplemental Table S2 (.xlsx, 1.5 MB) - Proteomic data II
- Supplemental Table S3 (.pdf, 66 KB) - Label Free Proteomic data ? Up-regulated proteins (u-A1) ? Ingenuity Analysis
- Supplemental Table S4 (.pdf, 66 KB) - Label Free Proteomic data ? Down-regulated proteins (d-A1) ? Ingenuity Analysis
- Supplemental Table S5 (.xlsx, 77 KB) - Label Free Proteomic data ? David Analysis
- Supplemental Table S6 (.pdf, 68 KB) - The cancer related information available in PubMed for HRS members.
- Supplemental Table S7 (.pdf, 80 KB) - Multivariate analyses to evaluate the independent prognostic value of dA1 and HRS.
- Supplemental Table S8 (.pdf, 87 KB) - Multivariate analyses to evaluate the independent prognostic value of KIFC1. LRRC59. and TRIP13.
- Supplemental Table S9 (.pdf, 74 KB) - Evaluation of over- and under-expression of KIFC1, LRRC59, and TRIP13 in cancer versus normal tissue within the Oncomine patient's dataset.
- Supplemental Figure S1 (.pdf, 1.0 MB) - HMGA1 silencing in MDA-MB-231 breast cancer cells causes a spindle-like fibroblastic/flattened and polygonal morphology transition reminiscent of a Mesenchymal/Epithelial transition.
- Supplemental Figure S2 (.pdf, 1.4 MB) - SDS-PAGE and western blot analysis to check the efficacy and reproducibility of siRNA-mediated HMGA1a silencing in MDA-MB-231 cells.
- Supplemental Figure S3 (.pdf, 556 KB) - Semi-quantitative western blot analysis for the evaluation of the level of HMGA1 silencing.
- Supplemental Figure S4 (.pdf, 414 KB) - Proteins whose expressions are inversely proportional to HMGA1 expression level (u-A1 protein set) do not represent a signature associated with clinical outcome of breast cancer patients and are not enriched in specific cancer subtypes.
- Supplemental Figure S5 (.pdf, 158 KB) - HRS members are component of several breast cancer-associated gene signatures.
- Supplemental Figure S6 (.pdf, 127 KB) - The expression of KIFC1, LRRC59, and TRIP13 is linked to HMGA1.
- Supplemental Figure S7 (.pdf, 6.3 MB) - HMGA1 positivity is linked to KIFC1, LRCC59, and TRIP13 positivity in breast cancer specimens.
- Supplemental Figure S8 (.pdf, 6.4 MB) - HMGA1 positivity is linked to KIFC1, LRCC59, and TRIP13 positivity in breast cancer specimens.
- Supplemental Figure S9 (.pdf, 20.5 MB) - Western blot analyses for the evaluation of antibody specificity.
- Supplemental Figure S10 (.pdf, 22.3 MB) - Western blot analyses for the evaluation of antibody specificity.
- Supplemental Figure S11 (.pdf, 15.7 MB) - The silencing of KIFC1, LRRC59, and TRIP13 causes a mesenchymal-epithelial morphological transition in MDA-MB-157 cells that is accompanied by a strong impairment of cell motility.
