# Supplementary material for: Translating Proteomic Into Functional Data: An High Mobility Group A1 (HMGA1) Proteomic Signature Has Prognostic Value in Breast Cancer
Source: Mol Cell Proteomics. 2015 Nov 2;15(1):109–23. doi: 10.1074/mcp.M115.050401 (PMC4762532; doi:10.1074/mcp.M115.050401)
Supplement: Supplemental Data [file 10.1074_M115.050401_mcp.M115.050401-1.pdf]

SUPPLEMENTAL FIGURE LEGENDS

Supplemental Figure S1: HMGA1 silencing in MDA-MB-231 breast cancer cells causes a spindle-like fibroblastic/flattened and polygonal morphology transition reminiscent of a Mesenchymal/Epithelial transition.

MDA-MB-231 cells have been treated with control- (left side - siCTRL) or HMGA1-targeting (right side – siA1_3) siRNA for 72 hours. Images have been captured on an inverted optical microscope. White bars indicate 100 μm.

Supplemental Figure S2: SDS-PAGE and western blot analysis to check the efficacy and reproducibility of siRNA-mediated HMGA1a silencing in MDA-MB-231 cells. MDA-MB-231 cells have been treated with control- (siCTRL) or HMGA1-targeting (siA1_3) siRNA for 72 hours. Cells have been lysed with SDS sample buffer and proteins analysed both by SDS-PAGE (staining by blue coomassie (CBB) to check normalization) and western blot (detection by α-HMGA1 antibody and normalization /transfer control by ponceau staining). Molecular weight markers (KDa) are indicated on the left.

Supplemental Figure S3: Semi-quantitative western blot analysis for the evaluation of the level of HMGA1 silencing. A: MDA-MB-231 cells have been treated with control- (siCTRL) or HMGA1-targeting (siA1_3) siRNA for 72 hours. Cells have been lysed with SDS sample buffer and proteins analysed by western blot (detection by α-HMGA1 antibody and normalization/transfer control by ponceau staining). 3.5 μg (lanes 1-6) and 1.75 μg (lanes 7-12) of each lysates was loaded. C1-C3: MDA-MB-231 cells treated with siCTRL; A1-A3: MDA-MB-231 cells treated with siA1_3. Molecular weight markers (KDa) are indicated on the left. B: Densitometric analysis of the western blot shown in (A). C: Histogram graph showing mean value and standard deviation relative to the densitometric analysis shown in (B).

Supplemental Figure S4: Proteins whose expressions are inversely proportional to HMGA1 expression level (u-A1 protein set) do not represent a signature associated with clinical outcome of breast cancer patients and are not enriched in specific cancer subtypes. A-B: Kaplan-Meier plots for Overall Survival (OS) and Recurrence Free Survival (RFS) with regard to the gene expression level (low or high) in a breast cancer gene expression meta-dataset (Kmplot collection v2014) of the set of proteins that are up regulated following HMGA1 silencing (u-A1 protein set). C-F: Gene Set Analysis (GSA) of the u-A1

protein set expression in a breast cancer gene expression meta-dataset (GOBO collection v2014). Box plots illustrating the expression distribution of u-A1 protein set across different cancer subtypes (Hu and PAM50 subtypes), ER negative and positive breast cancers, and breast cancers of different histological grade (1, 2, and 3). Numbers above the charts indicate the patients in each subtype group.

Supplemental figure S5: HRS members are component of several breast cancer-associated gene signatures. A: Breast cancer-associated gene signatures present in GeneSigDB were searched for the presence of HRS members. The presence of each HRS member within the indicated gene signature is evidenced by a red box. Signatures are listed according to the significativity of overlap with the HRS. B: Information regarding the analysed gene signatures.

Supplemental figure S6: The expression of KIFC1, LRRC59, and TRIP13 is linked to HMGA1. MDA-MB-157 cells were treated with control (siCTRL) or HMGA1-targeting siRNAs (siA1_1 and siA1_3) for 72 h. Protein expression levels of the indicated genes were analysed by western blot. Representative WB analyses are shown together with a red ponceau stained membrane to verify total protein normalization. The histogram graphs relative to western blot analyses were obtained using densitometric analyses (siCTRL vs. siA1_1 and siA1_3). The bars indicate the mean ± SD (n=3). Statistical significance was assessed with Student's t-test (*: $P < 0.05$; **: $P < 0.01$; ***: $P < 0.001$).

Supplemental figure S7: HMGA1 positivity is linked to KIFC1, LRCC59, and TRIP13 positivity in breast cancer specimens. Immunohistochemistry analyses performed on breast cancer specimens (ILC, invasive lobular carcinoma; IDC, invasive ductal carcinoma; DCIS, ductal carcinoma in situ) for the evaluation of KIFC1, LRRC59, TRIP13, and HMGA1 expression. Positivity is indicated by a red contour, negativity is indicated by a green contour. The antibodies used for these analyses are: KIFC1, AB172620; LRRC59, PA5–32057; TRIP13, HPA005727; HMGA1, homemade 1.

Supplemental figure S8: HMGA1 positivity is linked to KIFC1, LRCC59, and TRIP13 positivity in breast cancer specimens. Immunohistochemistry analyses performed on breast cancer specimens (ILC, invasive lobular carcinoma; IDC, invasive ductal carcinoma; DCIS, ductal carcinoma in situ) for the evaluation of KIFC1, LRRC59, TRIP13, and HMGA1 expression. Positivity is indicated by a red contour,

negativity is indicated by a green contour. The antibodies used for these analyses are: KIFC1, AB117535; LRRC59, HPA030827; TRIP13, HPA053093; HMGA1, homemade 2.

Supplemental figure S9: Western blot analyses for the evaluation of antibody specificity.

Different amounts (5, 10, and 20 μg) of MDA–MB–231 and MDA–MB–157 lysates were SDS–PAGE separated and western blot analysed using the indicated antibodies. Ponceau stained membrane are shown for the visualization of protein patterns. On the right side of each western blot analysis, a box shows an antibody recognition of lysates obtained from cells treated with control siRNA (siCTRL) or siRNA specific for the analysed protein (siHMGA1, siKIFC1, siLRRC59, or siTRI13). Molecular markers are indicated on the left (kDa). The antibodies tested are the following: Homemade 1 (HMGA1), ab172620 (KIFC1), PA5–32057 (LRRC59), and HPA005727 (TRIP13). These antibodies were used in the analyses shown in figure 5 and in supplemental Figure S7. All of them displayed a good SDS–PAGE/WB specificity. The anti–TRIP13 antibody gave a minor aspecific band but the major band corresponds to the expected molecular weight of TRIP13. The identities of the bands were confirmed by the use of specific siRNA molecules.

Supplemental figure S10: Western blot analyses for the evaluation of antibody specificity.

Different amounts (5, 10, and 20 μg) of MDA–MB–231 and MDA–MB–157 lysates were SDS–PAGE separated and western blot analysed using the indicated antibodies. Ponceau stained membrane are shown for the visualization of protein patterns. On the right side of each western blot analysis, a box shows an antibody recognition of lysates obtained from cells treated with control siRNA (siCTRL) or siRNA specific for the analysed protein (siHMGA1, siKIFC1, siLRRC59, or siTRI13). Molecular markers are indicated on the left (kDa). The antibodies tested are the following: Homemade 2 (HMGA1), ab117535 (KIFC1), HPA030827 (LRRC59), and HPA053093 (TRIP13). These antibodies were used in the analyses shown in supplemental Figure S8. The anti–HMGA1 antibody (homemade 2) displays a good SDS–PAGE/WB specificity. The anti–KIFC1 gave aspecific bands with intensity almost equal to the KIFC1 expected band. The anti–LRRC59 antibody gave minor aspecific bands but the major band corresponds to the expected molecular weight of LRRC59. The anti–TRIP13 antibody in the conditions and dilution (recommended by the manufacturer) used for SDS–PAGE/WB analyses displayed both a low sensitivity and specificity. The identities of the bands were confirmed by the use of specific siRNA molecules.

Supplemental figure S11: The silencing of KIFC1, LRRC59, and TRIP13 causes a mesenchymal-epithelial morphological transition in MDA-MB-157 cells that is accompanied by a strong impairment of cell motility. MDA-MB-157 cells were treated with control siRNA (siCTRL) or siRNA targeting KIFC1, LRRC59, and TRIP13 (siKIFC1, siLRRC59, and siTRIP13). After 72 h, the cells were evaluated for their morphology (optical microscope) (A) and motility using wound healing assays (B). The scale bar represents 50 μm. The bars indicate the mean ± SD (n=3, each point is a technical duplicate). Statistical significance was assessed with Student's t-test (*: $P < 0.05$; **: $P < 0.01$; ***: $P < 0.001$). The evaluation of silencing efficacy was performed by lysing the cells in SDS-sample buffer after the wound healing assay and analysing the expression of the three proteins by western blot. Representative WB analyses are shown together with red ponceau stained membranes to verify total protein normalization. Relative protein expression levels were obtained by densitometric analyses of WB analyses (siCTRL vs siKIFC1, siLRRC59, and siTRIP13). The bars indicate the mean ± SD (n=3). Statistical significance was assessed with Student's t-test (*: $P < 0.05$; **: $P < 0.01$; ***: $P < 0.001$).

Supplemental Experimental Procedures

siRNA sequences

Gene	*siRNA (5'-3')*
CTRL	ACAGUCGCGUUUGCGACUGTT
A1_1	GACAAGGCUAACAUCCCACTT
A1_3	ACUGGAGAAGGAGGAAGAGTT
TRIP13	CCCAUCGAUUUGAGUGCAUTT
KIFC1	GCCCAGAAUGAACGGUCAUTT
LRRC59	GGAUGGGACUGCAACUCAUTT

qRT–PCR primer sequences

Gene	***Forward*** (5'-3')	***Reverse*** (5'-3')
GAPDH	TCTCTGCTCCTCCTGTTC	GCCCAATACGACCAAATCC
HMGA1	ACCAGCGCCAAATGTTCATCCTCA	AGCCCCTCTTCCCCACAAAGAGT
TRIP13	TCAACAGCAACCTCATCACC	CAGGGATGTTTTTCCAGTGC
KIFC1	ATTACCACATCCCACCCAAG	TCTGGTTCTTCAACCCTGTG
LRRC59	TGAAGTGGTTGGACCTGAAG	ACACCTTGTTTGCACACTGC

Antibodies

Protein	***First SET***	***Second SET***
HMGA1	Homemade 1 (rabbit polyclonal)	Homemade 2 (rabbit polyclonal)
KIFC1	ab172620 (Abcam)	ab117535 (Abcam)
LRRC59	PA5–32057 (ThermoFisher/Pierce)	HPA030827 (SigmaAldrich)
TRIP13	HPA005727 (SigmaAldrich)	HPA053093 (SigmaAldrich)

Criteria for PubMed literature searches (Supplemental Table S6)

For each protein the entire set of Protein and Gene names deposited in the Protein Knowledgebase (UniProtKB) are reported (first line); in the second line the word used for PubMed searches are reported (note that some terms have been removed due to the matching with not related proteins/factors/information.

(Protein and Gene names in Protein Knowledgebase (UniProtKB)) => All
(Protein and Gene names in Protein Knowledgebase (UniProtKB)) and ("cancer" or "tumor" or "tumour" or "neoplastic") => Cancer

ATAD2
("ATPase family AAA domain-containing protein 2" or "AAA nuclear coregulator cancer-associated protein" or "ANCCA" or "ATAD2")

("ANCCA" or "ATAD2") and ("cancer" or "tumor" or "tumour" or "neoplastic")

BAZ1B
("WBSC10" or "WBSCR10" or "WBSCR9" or "WSTF" or "Tyrosine-protein kinase BAZ1B" or "Bromodomain adjacent to zinc finger domain protein 1B" or "Williams syndrome transcription factor" or "Williams-Beuren syndrome chromosomal region 10 protein" or "Williams-Beuren syndrome chromosomal region 9 protein" or "hWALp2" or "BAZ1B")

(("WBSCR9" or "WSTF" or "Williams syndrome transcription factor" or "BAZ1B") not "Wireless Strategy Task Force) and ("cancer" or "tumor" or "tumour" or "neoplastic")

COPS2
(("COP9 signalosome complex subunit 2" or "SGN2 Signalosome subunit 2" or "Alien homolog JAB1-containing signalosome subunit 2" or "Thyroid receptor-interacting protein 15" or "TR-interacting protein 15" or "TRIP-15" or "COPS2" or "CSN2" or "TRIP15") not casein)

(("Thyroid receptor-interacting protein 15" or "TRIP-15" or "COPS2" or "CSN2" or "TRIP15") not ("casein" not "milk" not "national spina bifida")) and ("cancer" or "tumor" or "tumour" or "neoplastic")

CSF-1
("Macrophage colony-stimulating factor 1" or "CSF-1" or "M-CSF" or "MCSF" or "Lanimostim" or "CSF1")

("Macrophage colony-stimulating factor 1" or "CSF-1" or "M-CSF" or "MCSF" or "CSF1") and ("cancer" or "tumor" or "tumour" or "neoplastic")

DDX18
("ATP-dependent RNA helicase DDX18" or "DEAD box protein 18" or "Myc-regulated DEAD box protein" or "MrDb" or "DDX18")

("MrDb" or "DDX18") and ("cancer" or "tumor" or "tumour" or "neoplastic")

GFPT1
("Glutamine--fructose-6-phosphate aminotransferase [isomerizing] 1" or "D-fructose-6-phosphate amidotransferase 1" or "Glutamine:fructose-6-phosphate amidotransferase 1" or "GFAT 1" or "GFAT1" or "Hexosephosphate aminotransferase 1" or "GFPT1" or "GFAT" or "GFPT")

("GFAT 1" or "GFAT1" or "GFPT1" or "GFAT" or "GFPT") not ("goal-focused") and ("cancer" or "tumor" or "tumour" or "neoplastic")

GNAI3
("Guanine nucleotide-binding protein G(k) subunit alpha" or "G(i) alpha-3" or "GNAI3")

("G(i) alpha-3" or "GNAI3") and ("cancer" or "tumor" or "tumour" or "neoplastic")

DLGAP5
("Disks large-associated protein 5" or "DAP-5" or "Discs large homolog 7" or "Disks large-associated protein DLG7" or "Hepatoma up-regulated protein" or "HURP" or "DLGAP5" or "DLG7" or "KIAA0008")

("DAP-5" or "Hepatoma up-regulated protein" or "HURP" or "DLGAP5" or "DLG7" or "KIAA0008") and ("cancer" or "tumor" or "tumour" or "neoplastic")

ILF2
("Interleukin enhancer-binding factor 2" or "Nuclear factor of activated T-cells 45 kDa" or "ILF2" or "NF45" or "PRO3063")

("Interleukin enhancer-binding factor 2" or "ILF2" or "NF45") and ("cancer" or "tumor" or "tumour" or "neoplastic")

KIF11
("Kinesin-like protein KIF11" or "Kinesin-like protein 1" or "Kinesin-like spindle protein HKSP" or "Kinesin-related motor protein Eg5" or "Thyroid receptor-interacting protein 5" or "TR-interacting protein 5" or "TRIP-5" or "KIF11" or "EG5" or "KNSL1" or "TRIP5")

(("Kinesin-like protein 1" or "KIF11" or "EG5" or "KNSL1") not ("cryoprotectants" or "noise dosimeters")) and ("cancer" or "tumor" or "tumour" or "neoplastic")

KIFC1
("Kinesin-like protein KIFC1" or "Kinesin-like protein 2" or "Kinesin-related protein HSET" or "KIFC1" or "HSET" or "KNSL2")

(("Kinesin-like protein 2" or "KIFC1" or "HSET" or "KNSL2") not ("education" not "TPX2")) and ("cancer" or "tumor" or "tumour" or "neoplastic")

LRRC59
("Leucine-rich repeat-containing protein 59" or "Ribosome-binding protein p34" or "p34" or "LRRC59" or "PRO1855")

(Ribosome-binding protein p34 or "LRRC59") and ("cancer" or "tumor" or "tumour" or "neoplastic")

NCAPG
("Condensin complex subunit 3" or "Chromosome-associated protein G" or "Condensin subunit CAP-G" or "hCAP-G" or "Melanoma antigen NY-MEL-3" or "Non-SMC condensin I complex subunit G" or "XCAP-G homolog" or "NCAPG" or "CAPG" or "NYMEL3")

(("hCAP-G" or "NCAPG" or "CAPG") not (instantaneous enthalpic growth capacity)) and ("cancer" or "tumor" or "tumour" or "neoplastic")

PGRMC1
("Membrane-associated progesterone receptor component 1" or "mPR" or "PGRMC1" or "HPR6.6" or "PGRMC")

("Membrane-associated progesterone receptor component 1" or "PGRMC1" or "HPR6.6" or "PGRMC") and ("cancer" or "tumor" or "tumour" or "neoplastic")

PRPF4B
("Serine/threonine-protein kinase PRP4 homolog" or "PRP4 kinase" or "PRP4 pre-mRNA-processing factor 4 homolog" or "PRPF4B" or "KIAA0536" or "PRP4" or "PRP4H" or "PRP4K")

("PRP4 kinase" or "PRPF4B" or "PRP4" or "PRP4K") and ("cancer" or "tumor" or "tumour" or "neoplastic")

RPRD1A
("Regulation of nuclear pre-mRNA domain-containing protein 1A" or "Cyclin-dependent kinase inhibitor 2B-related protein" or "p15INK4B-related protein" or "RPRD1A" or "P15RS")

("RPRD1A" or "P15RS") and ("cancer" or "tumor" or "tumour" or "neoplastic")

RRM2
("Ribonucleoside-diphosphate reductase subunit M2" or "Ribonucleotide reductase small chain" or "Ribonucleotide reductase small subunit" or "RRM2" or "RR2")

(("Ribonucleotide reductase small chain" or "Ribonucleotide reductase small subunit" or "RRM2") not (RNA recognition motif)) and ("cancer" or "tumor" or "tumour" or "neoplastic")

SMC2
("Structural maintenance of chromosomes protein 2" or "SMC protein 2" or "SMC-2" or "Chromosome-associated protein E" or "hCAP-E" or "XCAP-E homolog" or "SMC2" or "CAPE" or "SMC2L1" or "PRO0324")

("SMC-2" or "hCAP-E" or "SMC2" or "SMC2L1") and ("cancer" or "tumor" or "tumour" or "neoplastic")

TOP2A
("DNA topoisomerase 2-alpha" or "DNA topoisomerase II, alpha isozyme" or "TOP2A" or "TOP2")

("DNA topoisomerase 2-alpha" or "TOP2A" or "TOP2") and ("cancer" or "tumor" or "tumour" or "neoplastic")

TRIP13
("Pachytene checkpoint protein 2 homolog" or "Human papillomavirus type 16 E1 protein-binding protein" or "16E1-BP" or "HPV16 E1 protein-binding protein" or "Thyroid hormone receptor interactor 13" or "Thyroid receptor-interacting protein 13" or "TR-interacting protein 13" or "TRIP-13" or "TRIP13" or "PCH2")

(("16E1-BP" or "TRIP13" or "PCH2") not (Molybdenum or **polyester or** Pontocerebellar)) and ("cancer" or "tumor" or "tumour" or "neoplastic")

WHSC1
("Histone-lysine N-methyltransferase NSD2" or "Multiple myeloma SET domain-containing protein" or "MMSET" or "Nuclear SET domain-containing protein 2" or "NSD2" or "Protein trithorax-5 Wolf-Hirschhorn syndrome candidate 1 protein" or "WHSC1" or "WHSC1" or "KIAA1090" or "MMSET" or "NSD2" or "TRX5")

("MMSET" or "NSD2" or "WHSC1" or "WHSC1" or "MMSET" or "NSD2") and ("cancer" or "tumor" or "tumour" or "neoplastic")
