# Supplementary material for: Translating Proteomic Into Functional Data: An High Mobility Group A1 (HMGA1) Proteomic Signature Has Prognostic Value in Breast Cancer
Source: Mol Cell Proteomics. 2015 Nov 2;15(1):109–23. doi: 10.1074/mcp.M115.050401 (PMC4762532; doi:10.1074/mcp.M115.050401)
Supplement: Supplemental Data [file 10.1074_M115.050401_mcp.M115.050401-4.pdf]

Suppl. Table 3 – Label Free Proteomic data – Up-regulated proteins (u-A1) – Ingenuity Analysis		
**Diseases and Disorders**		
Name	***p-value***	***# Molecules***
Infectious Disease	3.18×10^{-04} - 2.82×10^{-02}	79
Dermatological Diseases and Conditions	4.72×10^{-04} - 2.82×10^{-02}	64
Immunological Disease	4.72×10^{-04} - 2.85×10^{-02}	25
Inflammatory Disease	4.72×10^{-04} - 2.82×10^{-02}	15
Developmental Disorder	6.90×10^{-04} - 2.82×10^{-02}	33
**Molecular and Cellular Functions**		
Name	***p-value***	***# Molecules***
Protein Synthesis	2.47×10^{-06} - 2.82×10^{-02}	40
Gene Expression	8.64×10^{-06} - 2.03×10^{-02}	18
Energy Production	5.42×10^{-05} - 1.10×10^{-02}	20
Lipid Metabolism	5.42×10^{-05} - 2.82×10^{-02}	37
Small Molecule Biochemistry	5.42×10^{-05} - 2.82×10^{-02}	74
**Physiological System Development and Function**		
Name	***p-value***	***# Molecules***
Behavior	7.92×10^{-04} - 2.82×10^{-02}	2
Hair and Skin Development and Function	2.33×10^{-03} - 2.82×10^{-02}	3
Tumor Morphology	5.15×10^{-03} - 2.82×10^{-02}	5
Renal and Urological System Development and Function	7.49×10^{-03} - 7.49×10^{-03}	2
Tissue Development	7.49×10^{-03} - 2.82×10^{-02}	7
