# Supplementary material for: Translating Proteomic Into Functional Data: An High Mobility Group A1 (HMGA1) Proteomic Signature Has Prognostic Value in Breast Cancer
Source: Mol Cell Proteomics. 2015 Nov 2;15(1):109–23. doi: 10.1074/mcp.M115.050401 (PMC4762532; doi:10.1074/mcp.M115.050401)
Supplement: Supplemental Data [file 10.1074_M115.050401_mcp.M115.050401-5.pdf]

Suppl. Table 4 – Label Free Proteomic data – Down-regulated proteins (d-A1) – Ingenuity Analysis		
**Diseases and Disorders**		
Name	***p-value***	***# Molecules***
Infectious Disease	1.48×10^{-06} - 2.75×10^{-02}	87
Organismal Injury and Abnormalities	2.63×10^{-05} - 2.75×10^{-02}	29
Dermatological Diseases and Conditions	2.76×10^{-05} - 2.75×10^{-02}	27
Renal and Urological Disease	4.46×10^{-05} - 2.75×10^{-02}	36
Cancer	1.93×10^{-04} - 2.75×10^{-02}	150
**Molecular and Cellular Functions**		
Name	***p-value***	***# Molecules***
Cell Cycle	5.27×10^{-10} - 2.75×10^{-02}	95
Cellular Movement	9.92×10^{-09} - 2.75×10^{-02}	59
Cellular Assembly and Organization	1.12×10^{-07} - 2.75×10^{-02}	69
DNA Replication, Recombination, and Repair	1.12×10^{-07} - 2.75×10^{-02}	65
Cell Signaling	1.14×10^{-06} - 2.75×10^{-02}	31
**Physiological System Development and Function**		
Name	***p-value***	***# Molecules***
Cardiovascular System Development and Function	5.19×10^{-05} - 2.48×10^{-02}	24
Skeletal and Muscular System Development and Function	1.97×10^{-04} - 2.75×10^{-02}	6
Tissue Development	1.97×10^{-04} - 2.75×10^{-02}	27
Tumor Morphology	6.63×10^{-04} - 2.75×10^{-02}	11
Hematological System Development and Function	7.53×10^{-04} - 2.75×10^{-02}	8
