# Supplementary material for: Translating Proteomic Into Functional Data: An High Mobility Group A1 (HMGA1) Proteomic Signature Has Prognostic Value in Breast Cancer
Source: Mol Cell Proteomics. 2015 Nov 2;15(1):109–23. doi: 10.1074/mcp.M115.050401 (PMC4762532; doi:10.1074/mcp.M115.050401)
Supplement: Supplemental Data [file 10.1074_M115.050401_mcp.M115.050401-7.pdf]

Suppl. table 6 – The cancer related information available in PubMed for HRS members.

Protein	Ratio siA1_3/siCTRL		PubMed*		Role in cancer	Link with clinical data
	Array	Proteomic	All	Cancer		
ATAD2	0.299	0.338	37	33	Green	Green
BAZ1B	0.300	0.365	69	14	Yellow	Yellow
COPS2	0.167	0.673	66	18	Yellow	Red
CSF-1	0.295	0.312	5775	2213	Green	Green
DDX18	0.291	0.697	11	4	Yellow	Red
GFPT1	0.170	0.759	143	14	Red	Yellow
GNAI3	0.314	0.566	98	12	Yellow	Yellow
DLGAP5	0.322	0.524	90	40	Green	Green
ILF2	0.228	0.755	68	15	Yellow	Yellow
KIF11[1]	0.290	0.467	556	218	Green	Green
KIFC1	0.327	0.570	81	17	Yellow	Yellow
LRRC59	0.096	0.631	11	3	Yellow	Red
NCAPG	0.132	0.709	129	42	Green	Green
PGRMC1	0.278	0.230	155	59	Green	Yellow
PRPF4B	0.344	0.699	65	5	Yellow	Red
RPRD1A	0.278	0.362	6	5	Yellow	Red
RRM2	0.292	0.540	259	154	Green	Green
SMC2	0.285	0.645	106	22	Yellow	Yellow
TOP2A	0.234	0.420	602	428	Green	Green
TRIP13	0.348	0.705	59	7	Yellow	Yellow
WHSC1	0.152	0.681	163	102	Green	Green

Green: well established
Yellow: not unambiguously established / few information available
Red: no information available
* the criteria for PubMed literature researches are reported in supplementary methods (data relative to October 2014)
