# Supplementary material for: Translating Proteomic Into Functional Data: An High Mobility Group A1 (HMGA1) Proteomic Signature Has Prognostic Value in Breast Cancer
Source: Mol Cell Proteomics. 2015 Nov 2;15(1):109–23. doi: 10.1074/mcp.M115.050401 (PMC4762532; doi:10.1074/mcp.M115.050401)
Supplement: Supplemental Data [file 10.1074_M115.050401_mcp.M115.050401-8.pdf]

Suppl. table 7 - Multivariate analyses to evaluate the independent prognostic value of dA1 and HRS.

	d-A1			*HRS*		
	HR	*95 % CI*	*p value*	*HR*	*95 % CI*	*p value*
**OS**						
**Size: >20 mm**	1.95	1.47-2.61	<0.00001	1.99	1.49-2.65	<0.00001
**Age: > 50**	1.5	1.12-2.01	0.006	1.48	1.1-1.98	0.008
**Grade: G3**	1.26	0.91-1.75	0.17	1.2	0.84-1.69	0.31
**Node status: neg**	0.47	0.35-0.62	1.12	0.47	0.35-0.62	<0.00001
**ER status: pos**	0.89	0.64-1.26	0.51	0.91	0.65-1.27	0.57
**Expr. level: low**	0.68	0.51-0.92	0.01	0.7	0.5-0.97	0.031
**RFS**						
**Size: >20 mm**	1.73	1.36-2.21	<0.00001	1.72	1.35-2.2	0.000013
**Age: > 50**	0.86	0.67-1.11	0.25	0.85	0.66-1.1	0.21
**Grade: G3**	1.03	0.78-1.37	0.82	0.9	0.67-1.2	0.46
**Node status: neg**	0.69	0.53-0.91	0.007	0.71	0.54-0.92	0.01
**ER status: pos**	0.85	0.62-1.16	0.3	0.89	0.65-1.22	0.46
**Expr. level: low**	0.7	0.55-0.91	0.006	0.56	0.43-0.73	0.000015
**DMFS**						
**Size: >20 mm**	1.18	0.89-1.58	0.25	1.17	0.88-1.57	0.27
**Age: > 50**	1.2	0.89-1.61	0.23	1.2	0.89-1.61	0.22
**Grade: G3**	1.7	1.24-2.32	0.0008	1.6	1.16-2.2	0.003
**Node status: neg**	0.6	0.42-0.86	0.005	0.62	0.44-0.89	0.008
**ER status: pos**	0.93	0.66-1.29	0.65	0.95	0.68-1.33	0.76
**Expr. level: low**	0.61	0.45-0.82	0.001	0.58	0.42-0.81	0.001
