# Supplementary material for: Translating Proteomic Into Functional Data: An High Mobility Group A1 (HMGA1) Proteomic Signature Has Prognostic Value in Breast Cancer
Source: Mol Cell Proteomics. 2015 Nov 2;15(1):109–23. doi: 10.1074/mcp.M115.050401 (PMC4762532; doi:10.1074/mcp.M115.050401)
Supplement: Supplemental Data [file 10.1074_M115.050401_mcp.M115.050401-9.pdf]

Suppl. table 8 - Multivariate analyses to evaluate the independent prognostic value of KIFC1. LRRC59. and TRIP13.

	KIFC1			*LRRC59*			*TRIP13*		
	HR	*95 % CI*	*p value*	*HR*	*95 % CI*	*p value*	*HR*	*95 % CI*	*p value*
**OS**									
**Size: >20 mm**	1.96	1.47-2.61	<0.00001	1.99	1.5-2.66	<0.00001	1.95	1.46-2.6	0.00001
**Age: > 50**	1.47	1.1-1.96	0.01	1.49	1.11-1.99	0.008	1.42	1.06-1.9	0.02
**Grade: G3**	1.21	0.88-1.68	0.24	1.32	0.95-1.84	0.1	1.11	0.79-1.57	0.55
**Node status: neg**	0.46	0.35-0.6	<0.00001	0.47	0.36-0.62	<0.00001	0.47	0.35-0.62	<0.00001
**ER status: pos**	0.88	0.63-1.23	0.46	0.85	0.61-1.19	0.35	0.91	0.65-1.27	0.58
**Expr. level: low**	0.62	0.46-0.84	0.002	0.83	0.62-1.11	0.2	0.6	0.44-0.82	0.001
**RFS**									
**Size: >20 mm**	1.68	1.32-2.14	0.00003	1.73	1.35-2.2	1.00E-05	1.74	1.36-2.22	0.00001
**Age: > 50**	0.83	0.65-1.07	0.16	0.85	0.66-1.09	0.2	0.79	0.61-1.03	0.08
**Grade: G3**	0.93	0.7-1.23	0.6	0.75	0.37-1.54	0.72	0.85	0.63-1.14	0.28
**Node status: neg**	0.72	0.55-0.94	0.02	0.82	0.33-2.05	0.004	0.71	0.54-0.93	0.01
**ER status: pos**	0.9	0.66-1.22	0.5	0.52	0.24-1.12	0.15	0.91	0.66-1.24	0.55
**Expr. level: low**	0.46	0.35-0.6	<0.00001	2.01	1.11-3.65	0.04	0.52	0.4-0.68	<0.00001
**DMFS**									
**Size: >20 mm**	1.13	0.85-1.52	0.4	1.15	0.86-1.55	0.33	1.22	0.91-1.63	0.18
**Age: > 50**	1.2	0.89-1.62	0.22	1.21	0.9-1.63	0.2	1.15	0.85-1.54	0.36
**Grade: G3**	1.77	1.31-2.4	0.0002	1.77	1.3-2.4	3.00E-04	1.47	1.07-2.03	0.02
**Node status: neg**	0.61	0.43-0.88	0.007	0.63	0.44-0.89	0.01	0.6	0.42-0.86	0.005
**ER status: pos**	0.88	0.64-1.23	0.46	0.86	0.62-1.19	0.36	0.93	0.67-1.3	0.69
**Expr. level: low**	0.66	0.49-0.89	0.007	0.67	0.5-0.89	0.006	0.51	0.37-0.71	0.00006
