# Supplementary material for: Translating Proteomic Into Functional Data: An High Mobility Group A1 (HMGA1) Proteomic Signature Has Prognostic Value in Breast Cancer
Source: Mol Cell Proteomics. 2015 Nov 2;15(1):109–23. doi: 10.1074/mcp.M115.050401 (PMC4762532; doi:10.1074/mcp.M115.050401)
Supplement: Supplemental Data [file 10.1074_M115.050401_mcp.M115.050401-10.pdf]

Suppl. Table 9 - Evaluation of over- and under-expression of KIFC1, LRRC59, and TRIP13 in cancer versus normal tissue within the Oncomine patient's dateset.

Cancer type	*Cancer vs Normal*					
	**KIFC1**		**LRRC59**		**TRIP13**	
Bladder	2				3	
Brain and CNS	4					1
Breast	11		3		6	1
Cervical	2				2	
Colorectal	3				15	
Esophageal					2	
Gastric	2				5	
Head and Neck	1		1		6	
Kidney			1			
Leukemia		2			1	3
Liver			1		4	
Lung	7				9	
Lymphoma	1				3	
Melanoma	2		1		1	
Myeloma						
Other	2		6		2	2
Ovarian	2				2	
Pancreatic					1	
Prostate	4				1	
Sarcoma					7	
Significant Unique Analyses	42	2	13	0	69	7
Total Unique Analyses	459		365		467	
