# Supplementary figures and images for: Translating Proteomic Into Functional Data: An High Mobility Group A1 (HMGA1) Proteomic Signature Has Prognostic Value in Breast Cancer
Source: Mol Cell Proteomics. 2015 Nov 2;15(1):109–23. doi: 10.1074/mcp.M115.050401 (PMC4762532; doi:10.1074/mcp.M115.050401)

# S. Figure 1 - Maurizio et al.

**siCTRL**

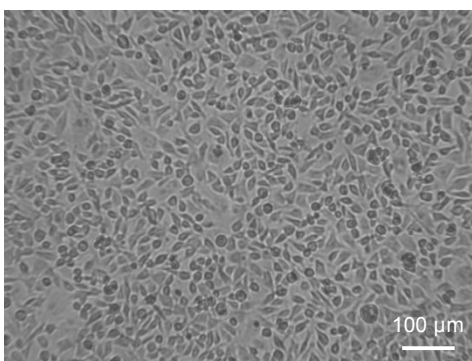

**siA1\_3**

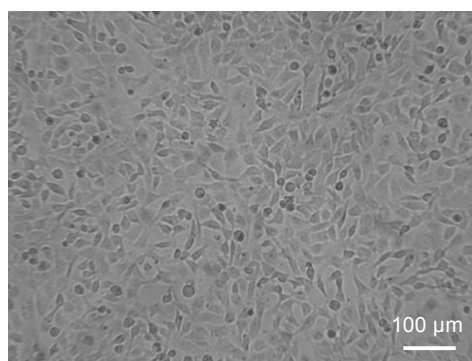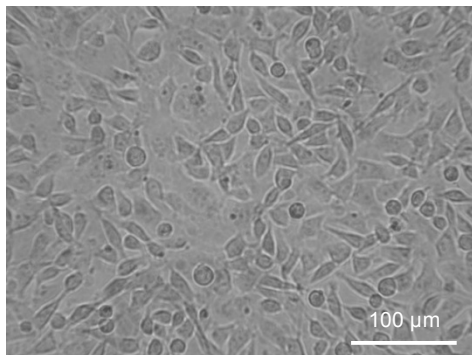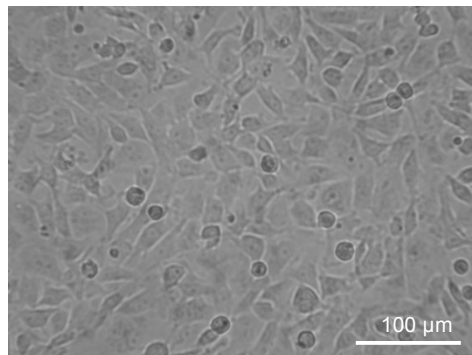

Supplement: Supplemental Data [file 10.1074_M115.050401_mcp.M115.050401-11.pdf]

S. Figure 2 - Maurizio et al.

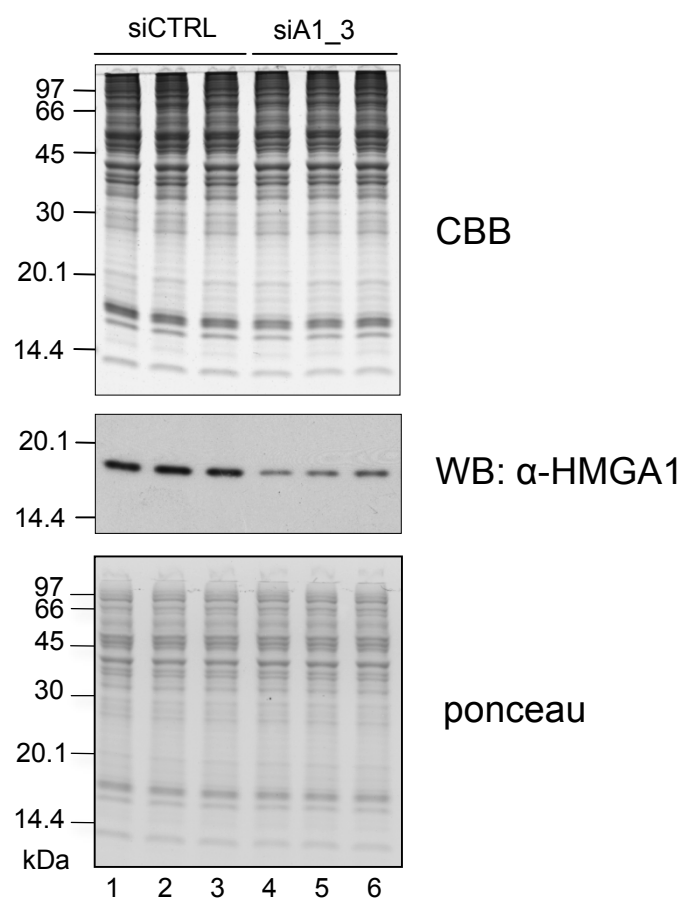

Supplement: Supplemental Data [file 10.1074_M115.050401_mcp.M115.050401-12.pdf]

# S. Figure 3 - Maurizio et al.

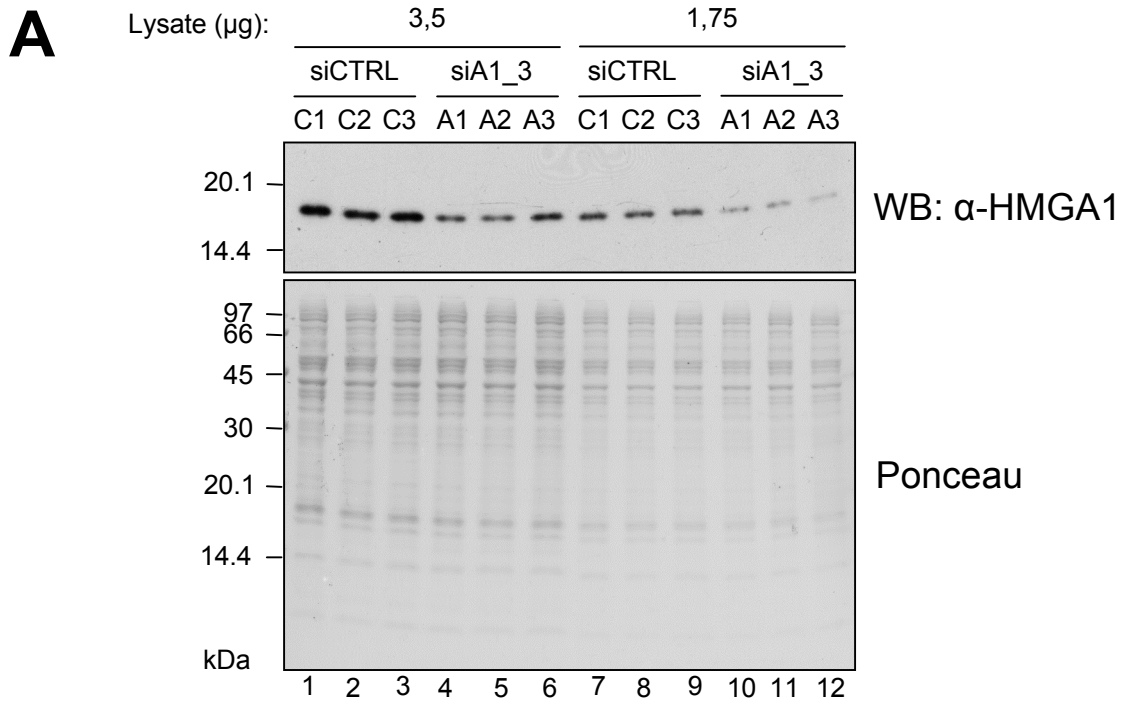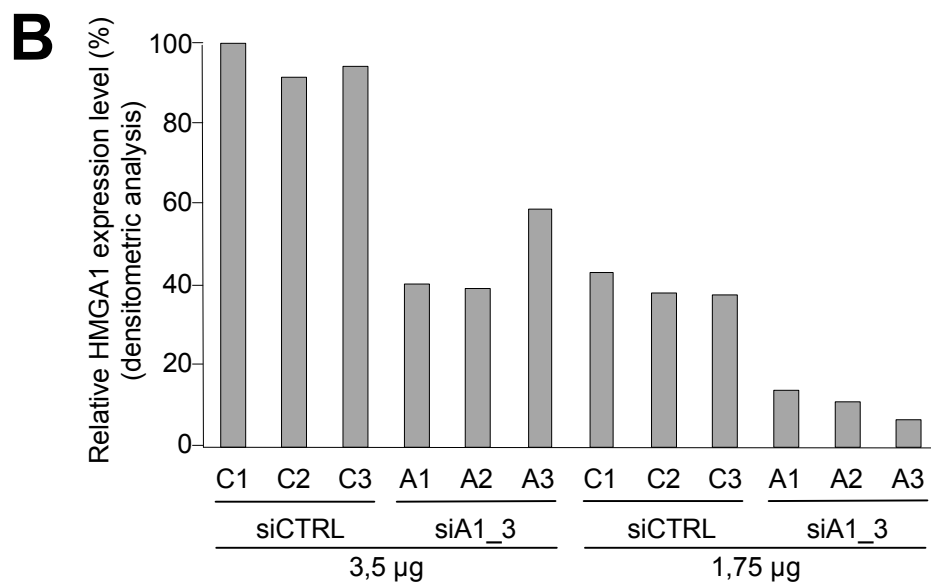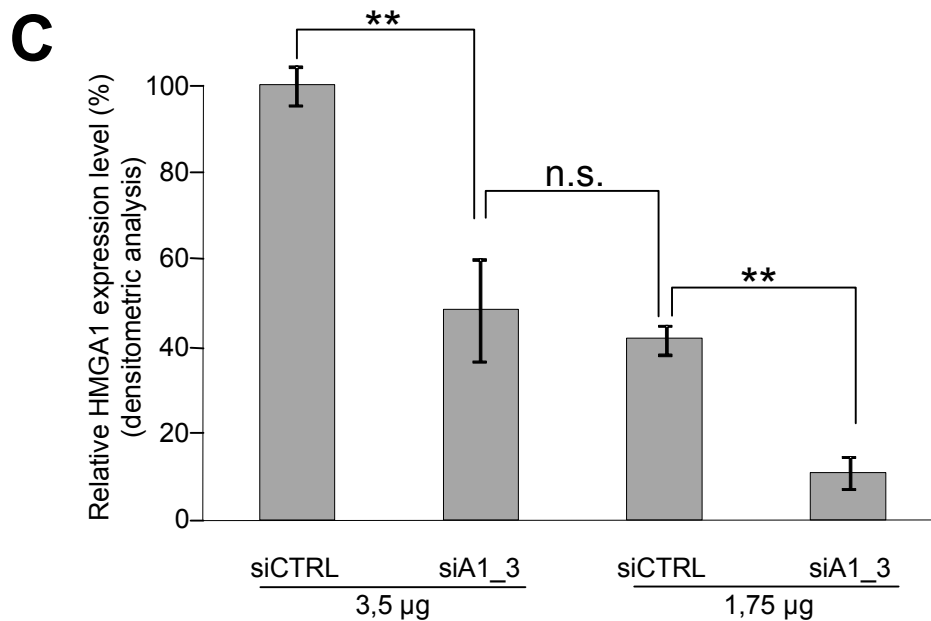

Supplement: Supplemental Data [file 10.1074_M115.050401_mcp.M115.050401-13.pdf]

S. Figure 4 - Maurizio et al.

u-A1

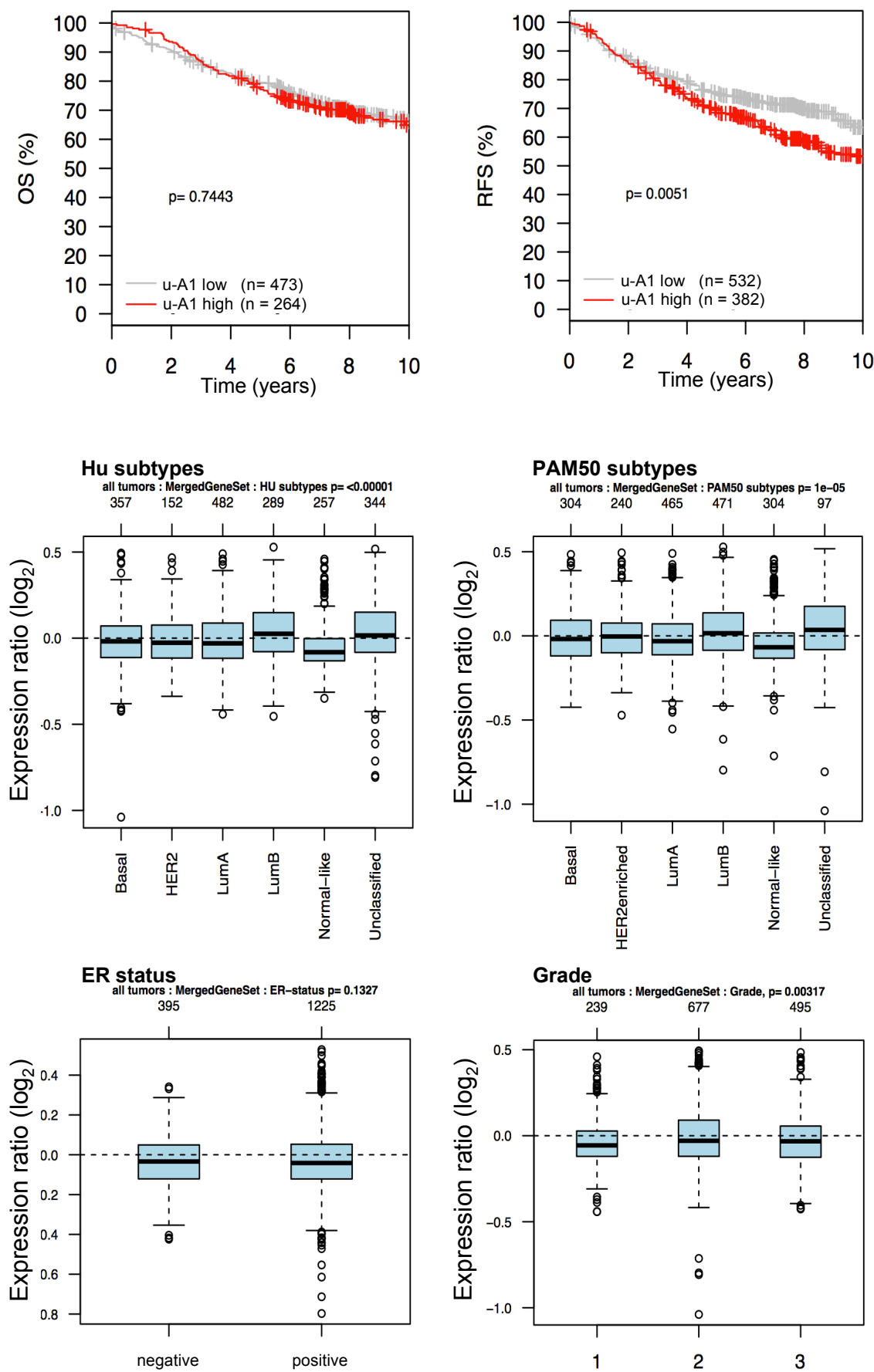

Supplement: Supplemental Data [file 10.1074_M115.050401_mcp.M115.050401-14.pdf]

S. Figure 6 - Maurizio et al.

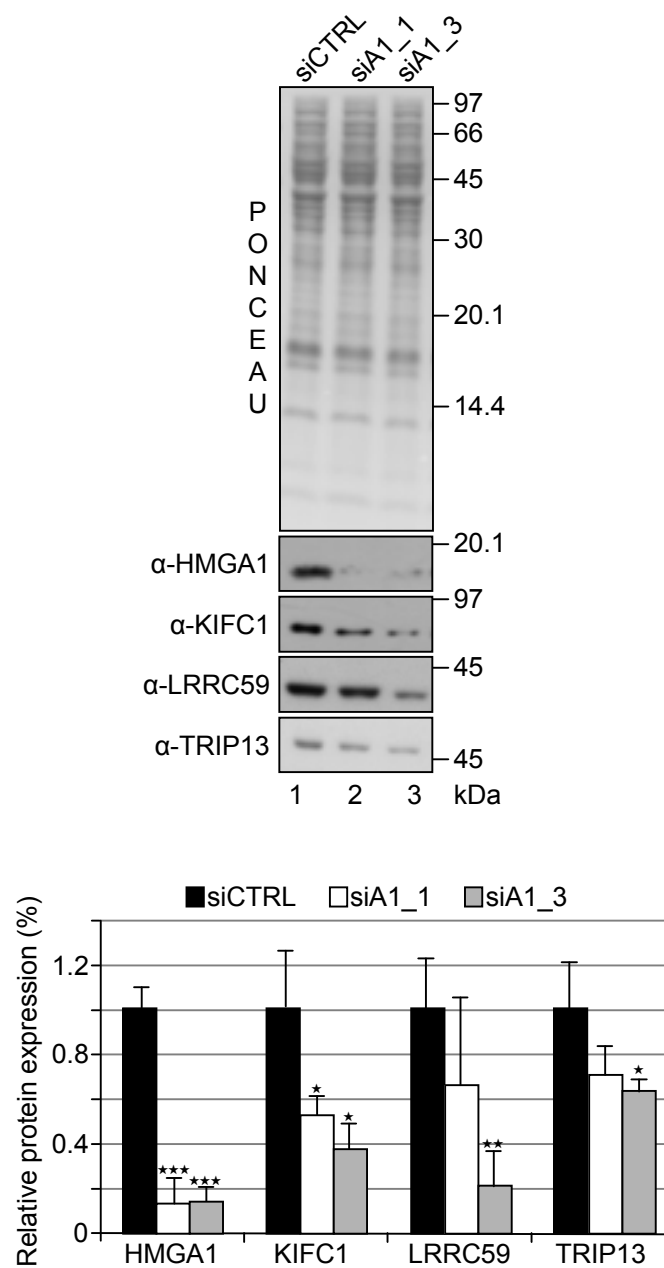

Supplement: Supplemental Data [file 10.1074_M115.050401_mcp.M115.050401-16.pdf]

## S. Figure 8 - Maurizio et al.

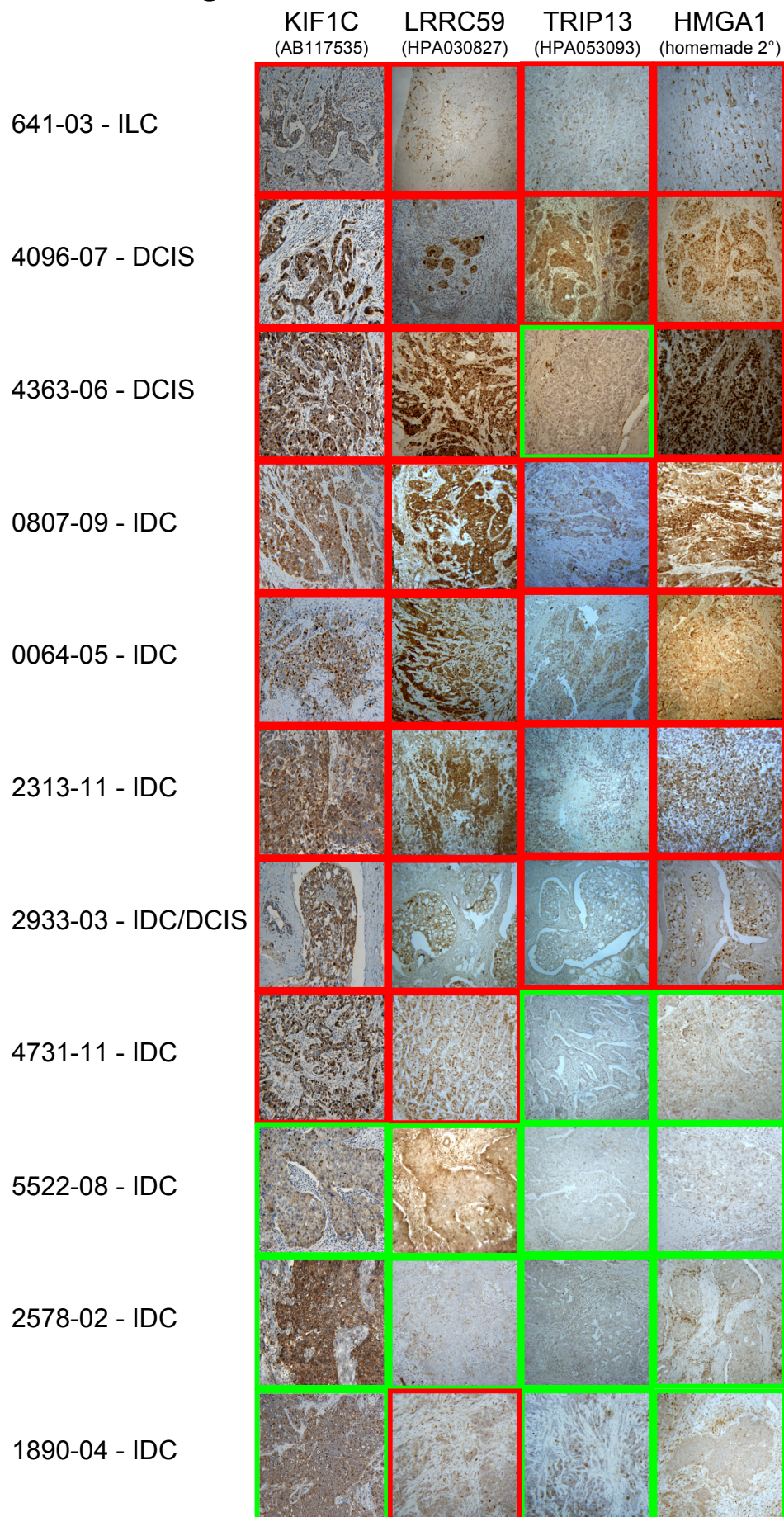

Supplement: Supplemental Data [file 10.1074_M115.050401_mcp.M115.050401-18.pdf]

S. Figure 9 - Maurizio et al.

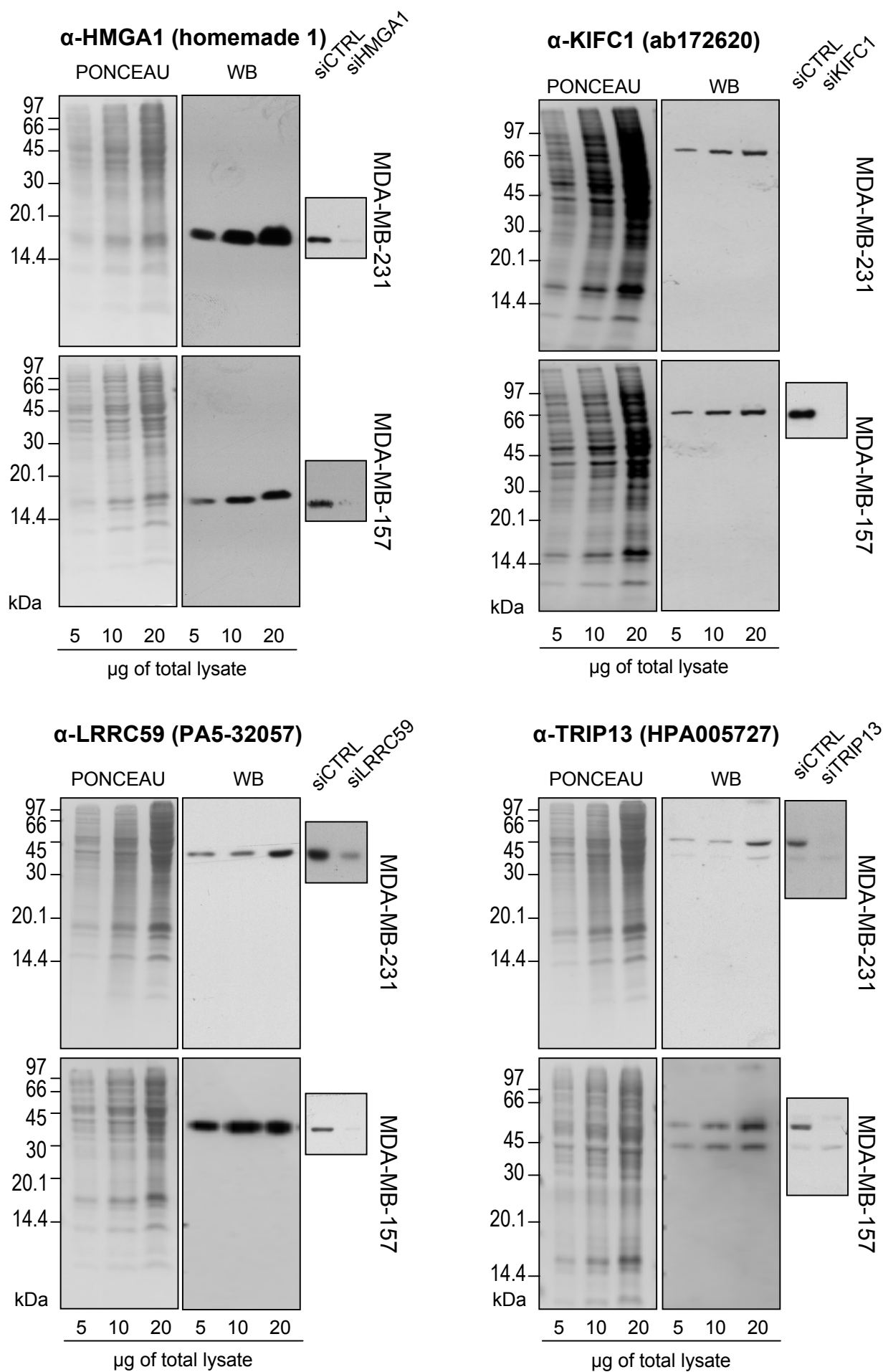

Supplement: Supplemental Data [file 10.1074_M115.050401_mcp.M115.050401-19.pdf]

S. Figure 10 - Maurizio et al.

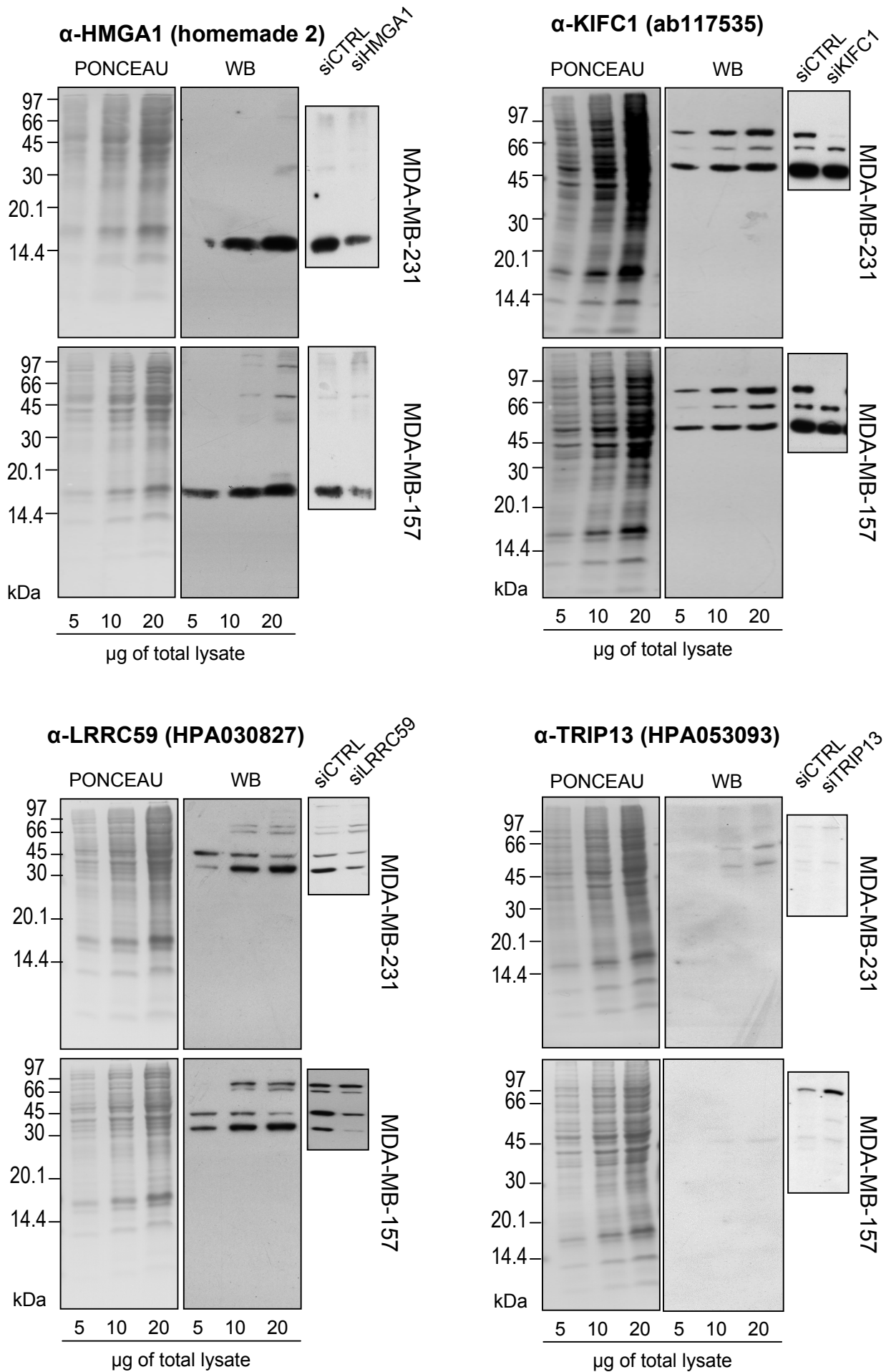

Supplement: Supplemental Data [file 10.1074_M115.050401_mcp.M115.050401-20.pdf]

# S. Figure 11 - Maurizio et al.

**A**

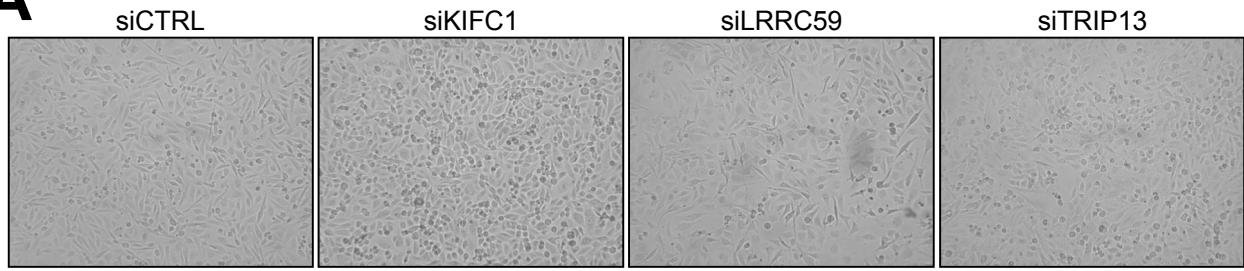

**B**

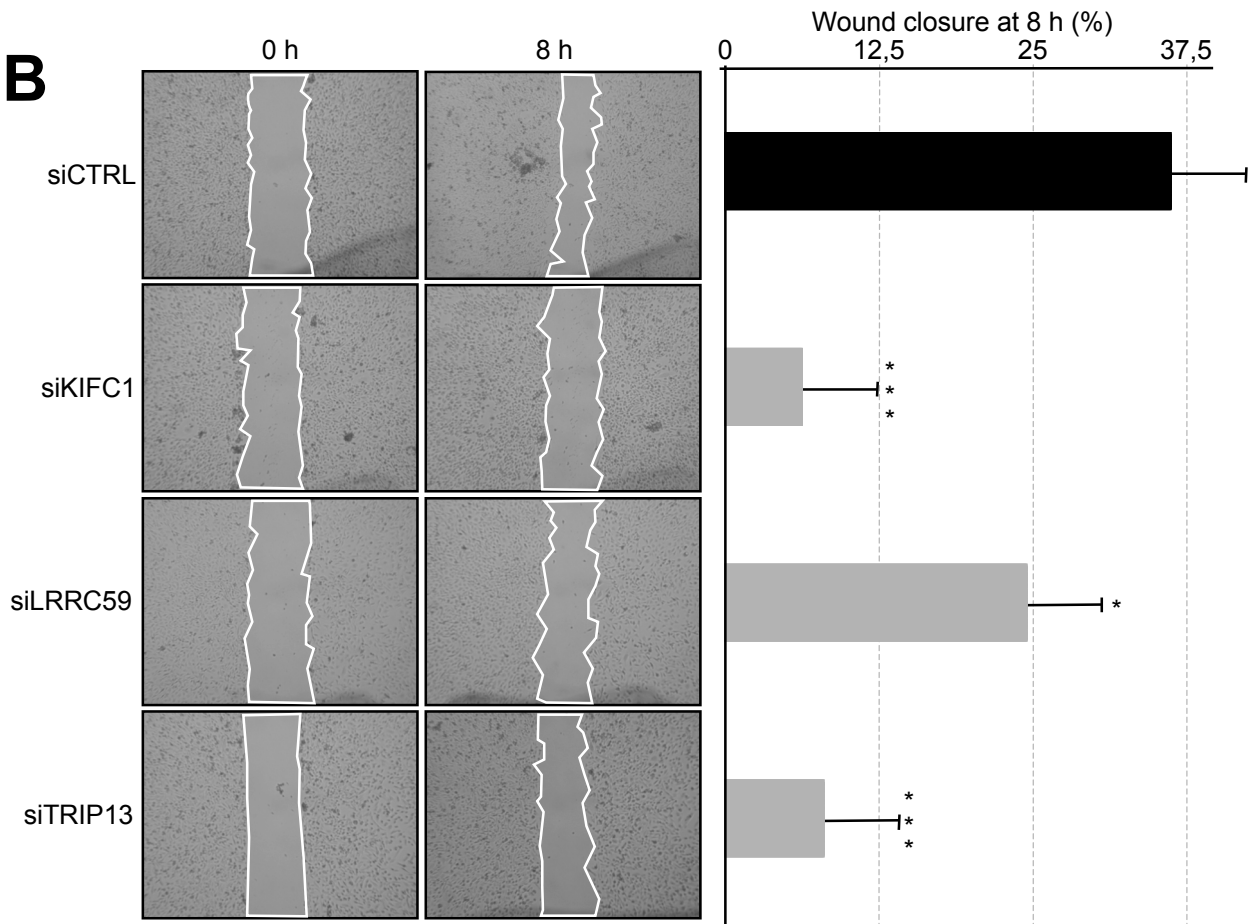

**C**

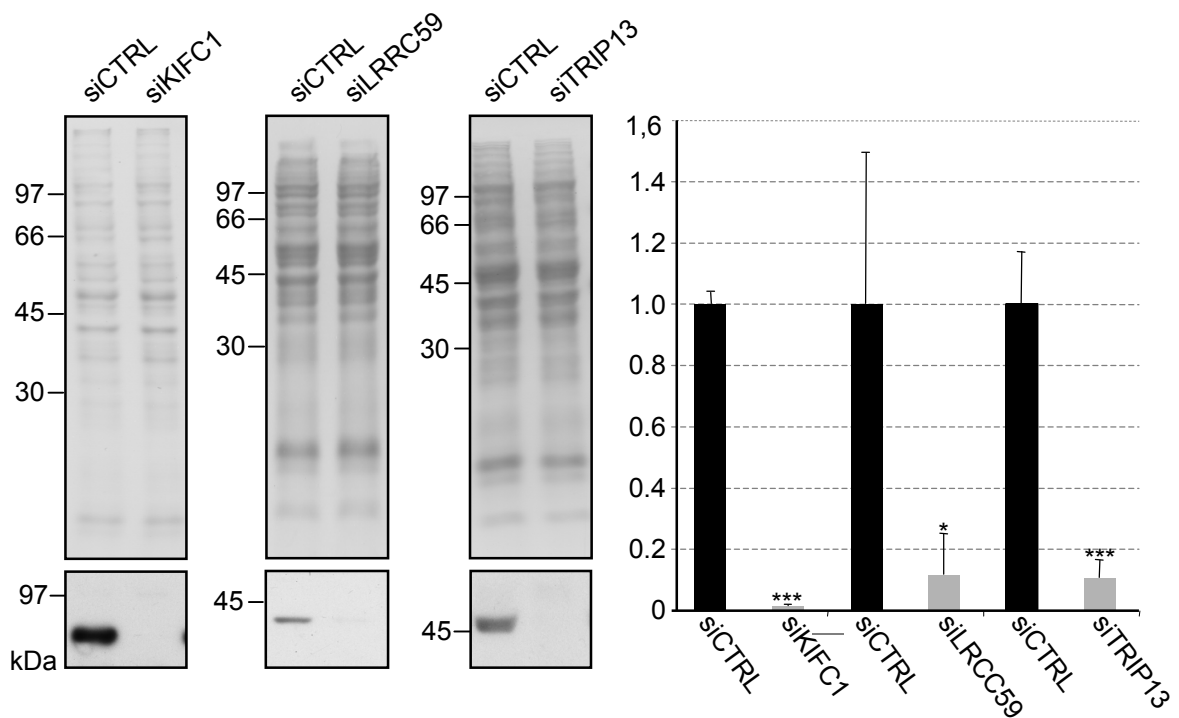

Supplement: Supplemental Data [file 10.1074_M115.050401_mcp.M115.050401-21.pdf]
