# Supplementary material for: Translating Proteomic Into Functional Data: An High Mobility Group A1 (HMGA1) Proteomic Signature Has Prognostic Value in Breast Cancer
Source: Mol Cell Proteomics. 2015 Nov 2;15(1):109–23. doi: 10.1074/mcp.M115.050401 (PMC4762532; doi:10.1074/mcp.M115.050401)
Supplement: Supplemental Data [file 10.1074_M115.050401_mcp.M115.050401-15.pdf]

S. Figure 5 - Maurizio et al.

A

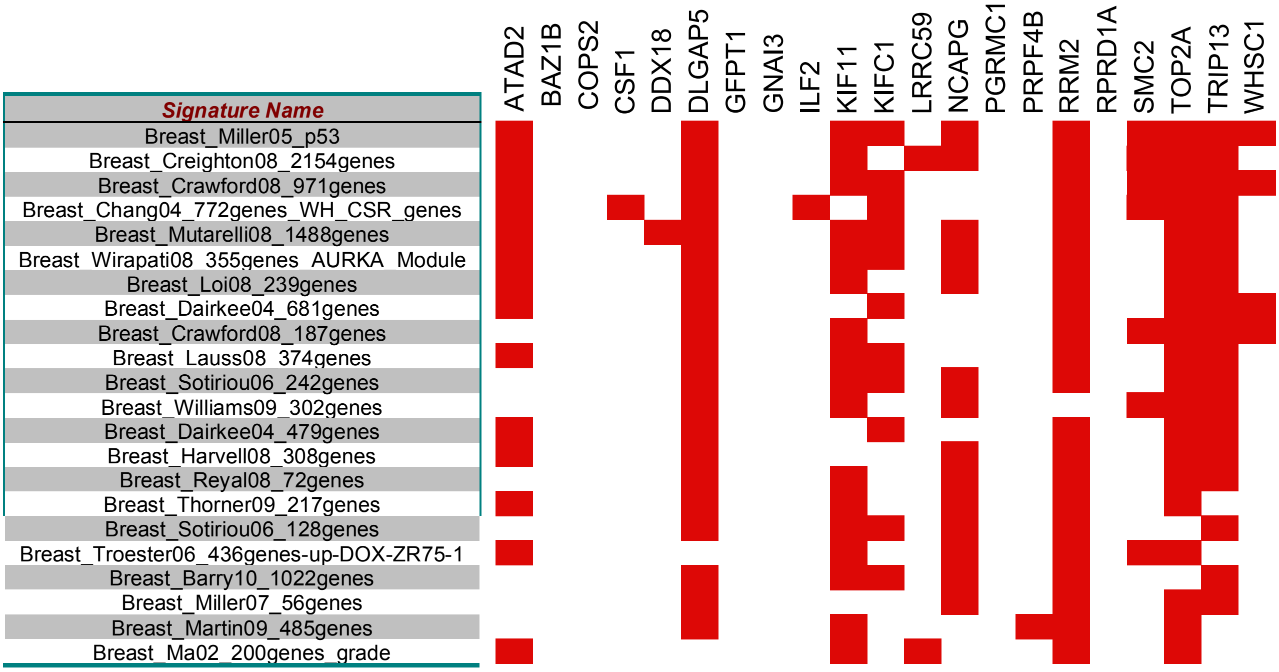

B

Signature Name	*Pubmed ID*	*Signature Size*	*Overlap*	*P*	*Argument*
Breast_Miller05_p53	16141321	868	10	0	p53 status and patient survival
Breast_Creighton08_2154genes	18757322	1803	9	0	Insulin-like growth factor-I and poor BC prognosis
Breast_Crawford08_971genes	18427120	376	9	0	Bromodomain 4 activation and BC survival
Breast_Chang04_772genes_WH_CSR_genes	14737219	589	9	0	fibroblast and human cancer progression
Breast_Mutarelli08_1488genes	18387200	1212	9	0	hormone-responsive human BC cells
Breast_Wirapati08_355genes_AURKA_Module	18662380	355	8	0	Meta-analysis of gene expression profiles in BC
Breast_Loi08_239genes	18498629	183	7	0	estrogen receptor-positive BC with tamoxifen
Breast_Dairkee04_681genes	15260889	523	7	0	primary BC cultures
Breast_Crawford08_187genes	18427120	141	7	0	Bromodomain 4 activation and BC survival
Breast_Lauss08_374genes	17899371	373	7	0	Consensus of literature to predict BC recurrence
Breast_Sotiriou06_242genes	16478745	205	7	0	Gene expression profiling in BC
Breast_Williams09_302genes	19798054	246	6	0	AP-2gamma in breast tumour cells
Breast_Dairkee04_479genes	15260889	378	6	0	primary BC cultures
Breast_Harvell08_308genes	18338247	215	6	0	Estrogen in resp to neoadjuvant endocrine therapy of BC
Breast_Reyal08_72genes	19014521	63	6	0	prognostic signatures in BC
Breast_Thorner09_217genes	19043454	176	6	0	B-Myb in basal-like BC
Breast_Sotiriou06_128genes	16478745	110	6	0	expression profiling in BC
Breast_Troester06_436genes-up-DOX-ZR75-1	17150101	342	6	0	p53 status in BC
Breast_Barry10_1022genes	20368555	850	6	0	Intratumor heterogeneity and BC biology and clinical outcome
Breast_Miller07_56genes	17885619	53	5	0	BC and letrozole
Breast_Martin09_485genes	19225562	428	5	0	tumor microenvironment between A/A and E/A BC patient
Breast_Ma02_200genes_grade	12714683	174	5	0	Gene expression profiles of human BC progression
